# Acknowledgement to Reviewers of *Materials* in 2019

**DOI:** 10.3390/ma13020482

**Published:** 2020-01-19

**Authors:** Materials Editorial Office

**Affiliations:** MDPI, St. Alban-Anlage 66, 4052 Basel, Switzerland

The editorial team greatly appreciates the reviewers who have dedicated their considerable time and expertise to the journal’s rigorous editorial process over the past 12 months, regardless of whether the papers are finally published or not. In 2019, a total of 4254 papers were published in the journal, with a median time to first decision of 14.82 days and a median time from submission to publication of 33 days. The editors would like to express their sincere gratitude to the following reviewers for their generous contribution in 2019:
A. Hof, LucasLiu, Ting-YuAbadal, SergiLiu, ZhixiaAbali, Emek B.Liu, XiangshengAbambres, MiguelLiu, Chao-LinAbate, LorenzoLiu, YaolongAbbas, AbdelgadirLiu, ZeliangAbbas, Tariq O.Liu, TianyuAbbaspour Tamijani, AliLiu, JinmingAbbina, SrinivasLiu, XingchenAbdelhafeez, Ali M.Liu, ManingAbdelkader, AmrLiu, YingtaoAbdellatef, MohammedLiu, Han YinAbdel-Mohti, AhmedLiu, XianhuAbdel-Motaleb, Ibrahim M.Liu, JingfeiAbdelrahman, MohamedLiu, Shyh-JiunAbdollahnejad, ZahraLiu, QiangAbdul Jabbar, Mohammed HussainLiu, Tzong-shiAbdul-Aziz, AliLiu, HuolongAbdullah, Nur AzamLiu, XiaoxiAbdullah, Oday IbraheemLiu, YaodongAbdulstaar, Mustafa A.Liu, WeiAbduo, JaafarLiu, XiaoAbdur, RahimLiu, ChongyangAbedi, RezaLiu, FeiAbendroth, BarbaraLiu, Yu-JingAbisset-Chavanne, EmmanuelleLiu, YongliangAbolfazl, ZahediLiudmila, RadionovaAboudi, JacobLivieri, PaoloAboulfadl, HishamLivieris, Ioannis E.Abousnina, RajabLiVoti, RobertoAbramchuk, MykolaLizundia, ErlantzAbramovich, HaimLlena, Carmen C.Abrams, StephenLlera, MiguelAbrate, SergeLloret, FernandoAbu-Dief, Ahmed M.Lloyd, Jeffrey T.Abuillan, WasimLo, Kuang YaoAbu-Niaaj, LubnaLo Frano, RosaAbu-Reziq, RaedLo Giudice, GiuseppeAbuzaid, AhmedLo Monaco, AngelaAcar, PınarLöbel, MartinAccardo, GraziaLobo, Rui F. M.Accorsi, GianlucaLocardi, FedericoAcha, VictorLocatelli, MarcelloAchchaq, FouziaLocci, Antonio MarioAchelle, SylvainLodyga-Chruscinska, ElżbietaAchilias, Dimitris S.Lofland, SamAchim, CristinaLoginova, IrinaAchyuthan, Komandoor E.Logoń, DominikAciu, ClaudiuLogozzo, SilviaAcker, JörgLoja, M.A.R.Adachi, ShinichiroLoka, ChadrasekharAdamcová, DanaLonardo, AmedeoAdamczyk-Habrajska, MałgorzataLoncar, JosipAdamus, GrażynaLongana, Marco L.Adarraga, ItziarLonghi, MariangelaAddou, RafikLongstaffe, James G.Adesina, AdeyemiLopez, FrancescoAdhikary, Suman KumarLopez, GartzenAdmal, NikhilLópez, ConcepciónAdonin, Sergey A.López Antón, RicardoAdrian, HenrykLópez de Lacalle, Luis NorbertoAdrover, AlessandraLopez Gayarre, FernandoAdusei, Paa KwasiLópez-Colina, CarlosAffolter, ChristianLópez-Estrada, LuisAfify, Ahmed SabryLópez-Fonseca, RubénAfify, AhmedLopez-Galilea, InmaculadaAflori, MagdalenaLópez-Garzón, F.J.Afrashtehfar, Kelvin IanLopez-Jaramillo, Francisco JavierAfsharipour, ElnazLópez-Lorente, Ángela InmaculadaAfshinnia, KavehLopez-Píriz, RobertoAgarwal, VivekLopez-Rodriguez, GemaAgathokleous, EvgeniosLópez-Salas, NievesAgeev, EduardLoprencipe, GiuseppeAggelis, Dimitrios G.Lord, OliverAgrawal, RichaLorena, Lorena FreitasAgrela, FranciscoLorenz, AlexanderÁgueda, Vicente I.Lorenz, MichaelAgüero, AlinaLorenzi, AndreaAguiar, JefferyLorenzi, SergioAguirre, RodolfoLorenzo, MiguelAgustina, AsenjoLorusso, MassimoAgustin-Panadero, RubenLosada-Perez, PatriciaAhamed Khan, Riaz AhamedLöser, WolfgangAhlswede, ErikŁosiewicz, BożenaAhmad, Hafiz WaqarLou, ShuaiAhmadi, MohammadaliLouarn, EssylltAhmadi, FarzadLouhenkilpi, SeppoAhmadivand, ArashLoures, LuisAhmed, FaridLovric, DinoAhmed, Hawreen HasanLow, It-Meng (Jim)Ahmed, Abubeker WorakeLowe, TerryAhmmed, K.M. TanvirLozano, DiegoAhn, KyunghanLozano-Guerrero, Antonio JoséAhn, Byung TaeLu, FeiAhn, Jin-SooLu, JingzhiAhn, ByungminLu, YubingAhsan, Shamim Md.Lu, YaoAhuir-Torres, JuanLu, ChenyangAikawa, ShinyaLu, ZiXingAimi, AkihisaLu, XuekunAiroudj, AissamLu, FrankAkagi, YukiLubchenko, VassiliyAkasaka, ShuichiLucaciu, Constantin MihaiAkazawa, HouseiLucchi, ElenaAkbari Garakani, MohammadLuciani, PaolaAkhavan, BehnamLucklum, FriederAkhnoukh, Amin K.Luda, Maria PaolaAkinjide, OluwajobiLufrano, FrancescoAkita, SadanoriLugoda, PasinduAkitsu, TakashiroLuk, Ting S.Ala, GuidoLukáč, PavelAl-Ajrash, SajaLukács, JánosAlaluss, KhaledLukaszewicz, AndrzejAl-Ameri, RiyadhLukinavicius, GrazvydasAl-Anssari, SarmadLukomska-Szymanska, MonikaAlar, VesnaLukoševičienė, OnaAlarco, JoseŁukowski, PawełAlatalo, Sara-MaariaLukoyanov, AlexeyAlba, MariaLukyanov, PavelAlbarracín, RicardoLunev, ArtemAl-Bataineh, SameerLuo, XiangangAlberti, Marcos G.Luo, LiangAlberto, NéliaLuo, NingqiAlbizuri, JosebaLuo, JianAlbu, MihaelaLuo, RongcongAlcock, BenLuong, Dzung DinhAlcoutlabi, MatazLupi, Saturnino MarcoAldazabal, JavierLupu, NicoletaAldemir-Bektas, BasakLussier, AaronAlderliesten, RenéLutsyk, PetroAldwell, BarryLuttenberger, Lidija RunkoAlegria, Elisabete C.B.A.Lutz, AlexanderAleksander, MucLuukkonen, TeroAleksandrova, MariyaLuzi, FrancescaAlemán, CarlosLv, YongGangAlemayehu, Fisseha M.Lv, ShenghuaAlessi, AntoninoLy, Thuc HueAlessio, VernaLykidis, CharalamposAlexander, BruceLyng, FionaAlexandrov, SergeiLyu, HailongAlexey, BeskopylnyLyulin, Alexey V.Alexiou, ChristophM. Shimpi, TusharAlfano, BrigidaM. Vorob'ev, MikhailAl-Fatimi, MohamedNaser, M.Z. Alfinito, EleonoraMa, JianminAlhussein, AkramMa, TianyiAl-Hussein, AbdullahMa, XiangAli, UsmanMa, LiangAli, YahiaMa, ChuyingAli Ansari, SajidMa, SunyoungAlicja, Kazek-KęsikMa, WanshuAlifui-Segbaya, FrankMaalouf, ChadiAlikin, DenisMaamoun, AhmedAlique, DavidMaazoun, AzerAljitawi, Omar S.Macdonald, DigbyAllais, FlorentMachno, MagdalenaAllegre, Olivier J.MacKenzie, Kenneth J.D.Alloza, IraideMackie, AllisonAl-Mamun, M.Mackiewicz, PiotrAlMangour, BandarMacQuarrie, StephanieAlmazan Lazaro, Juan AntonioMadaghiele, MartaAlmeida, Bernardo GonçalvesMadala, Hanumantha RaoAlmeida, FilipeMaddalena, RiccardoAlmeida Junior, Jose HumbertoMaddalone, MarcelloAlmoazen, HassenMadden, DavidAlnaser, Ali SamiMaddio, Pietro DavideAl-Obaidi, HishamMadej, DominikaAlosmanov, RasimMadenci, ErdoganAl-Qutaifi, SarahMadruga, SantiagoAl-Rahmani, AhmedMaeda, TomokiAl-Saadi, SaadMaekawa, KoichiAl-Sibahy, AdnanMagalhães, Fernão D.Altarawneh, MohammednoorMagdziak, MarekAltomare, LinaMagkoev, Tamerlan T.Alvarez, JoséMagna, GabrieleÁlvarez, PalomaMagnabosco, GiuliaÁlvarez, José AlbertoMagnani, MauroÁlvarez, ManuelMagyari, KlaraÁlvarez-Alonso, PabloMahapatra, Manoj K.Álvaro, CaballeroMahata, Manoj KumarAlves de Sousa, Ricardo J.Mahdi, MoinulAmalu, Emeka H.Mahedi, MasrurAmarande, LuminitaMahesh, SachithanadamAmarowicz, RyszardMahmoud, AbdelfattahAmbu, RitaMahmud, Md ArafatAmeloot, MarcelMahy, Julien G.Amer, MohMaia, Lino M.Amine, MiledMaier, PetraAmini, ShahrouzMaier, CatalinaAmiri, Iraj SadeghMaineult, AlexisAmiri, LeylaMaior, IoanaAmirov, AbdulkarimMaiorana, CarloAmit, KumarMaiwa, HiroshiAmoako, KagyaMaj, PiotrAmores, MarcoMajak, JüriAmza, CatalinMajeed, FarhatAn, ChunjiangMajerníková, JankaAn, HyosungMajlinger, KornelAnanth, AntonyMajoinen, JohannaAnanthakrishnan, Soundaram J.Majumder, PoulamiAnastasiou, AntoniosMakeeva, Irina MikhailovnaAnastasiou, E.K.Mäki-Arvela, PäiviAndersson, BengtMakisha, NikolayAndersson, NilsMaksimkin, AlekseyAndisheh, KavehMakvandi, PooyanAndreev, Dmitrii E.Malachowski, JerzyAndreev, KonstantinMalaiškienė, JurgitaAndreola, FernandaMalakhov, DmitriAndreoli, EnricoMalandrino, GraziellaAndrew, NGO Chun YongMalard, BenoitAndrew, BrittanMalchik, FyodorAndrew, Trisha L.Malekan, MohammadAndrews, JosephMalik, Muhammad ImranAndrey V., EmelyanovMalikov, AlexanderAndrojić, IvicaMalinowski, Paweł H.Andryushin, Konstantin P.Malinowski, SzymonAndrzejewska, EwaMalka, DrorAndrzejewska, AngelaMalkowski, PiotrAndrzejewski, JacekMallavia, RicardoAngani, Chandra SekharMallineni, Sreekanth KumarAnggraini, Sri AyuMalta, GraziaAnghelina, Florina VioletaMalucelli, GiulioAngione, Maria DanielaMalvoni, MariaAngst, UeliMamiński, MariuszAngurel, L.A.Mamrashev, AlexanderAnimitsa, IrinaMan, AdrianAnita Ioana, VisanManabe, KeiAnnamalai, LeelavathiManakhov, AntonAnni, MarcoManakil, JaneAnnick, RubbensManas, MiroslavAnsermet, Jean-PhilippeManca, Pier PaoloAnthony, BenManceau, Jean-FrançoisAntoine, RodolpheMancini, SimoneAntoine, Claire Z.Manconi, MariaAntonaci, PaolaMandal, TirthaAntoniac, IulianMandić, VilkoAntonio, FormisanoManera, Maria GraziaAntonucci, Pier LuigiManfredi, LuigiAntony, JaneManfrinetti, PietroAntonyuk, SergiyMang, TiaAntoszewski, BogdanMangalara, Jayachandra HariAntunes, ElsaMangalathu, SujithAntunović, MajaMangano, FrancescoAnupam, KumarMania, SzymonAnuta, ValentinaManiatty, Antoinette M.Anyszka, RafałManière, CharlesAoh, Jong-NingManin, LionelAoki, HiroyukiMankey, GaryAoyagi, KentaMannino, SaverioAparicio, PatriciaMannu, AlbertoApetrei, ConstantinManolakos, DimitriosApicella, AntonioMante, Francis K.Apostol, MarianManthina, VenkataAquilano, DinoMao, YunweiArabzadeh, AliMara, DimitrijeAradilla, DavidMaragoni, LucioArafa, AhmedMarakatti, VijaykumarAramendia, IñigoMarasso, Simone LuigiAramesh, MortezaMarat-Mendes, RosaAranda, MiguelMaraveas, ChrysanthosAraujo, DanielMarcellini, MorenoAraújo, MariaMarcelot, OlivierAraújo-Gomes, NunoMarchenkov, ArtemArbenin, Andrei Y.Marchesan, SilviaArchodoulaki, Vasiliki-MariaMarchese, GiulioArczewska, MartaMarchetti, MarioArdashev, DmitriiMarchiori, Cleber F.N.Ardelean, Lavinia CosminaMarcian, PetrArenas, Luis FernandoMarcinauskas, LiutaurasArgilaga, AlbertMarco, PetroloArgitis, PanagiotisMarconcini, PaoloArgiz, CristinaMarenzi, GaetanoArgyle, MorrisMaria, TimofeevaArias-Pardilla, JoaquínMariani, StefanoArif, KhalidMarić, MilanAriga, KatsuhikoMarin, LuminitaArima, TakahisaMarinel, SylvainArima, HirofumiMarín-Genescà, MarcArjunan, ArunMarinić-Kragić, IvoArkadiusz, DziedzicMarinkovic, DraganArmentani, EnricoMarino, GiuseppeArmstrong, Ronald W.Marín-Ramos, Nagore I.Arnold, Donna C.Marinucci, LorellaArnusch, Christopher J.Marius, BodorArora, HimanshuMarjanović, MiroslavArrais, AldoMárk, Géza IstvánArrieta, Marina P.Markopoulos, AngelosArrigo, RossellaMarkvicka, EricArsenio, PedroMarmy, PierreArshad, AdeelMarohnic, TeaArtemev, AndreiMaroni, FabioArtigas, AlbertMarotti De Sciarra, FrancescoArtini, CristinaMarousek, JosefArtiukhina, EkaterinaMarpu, Sreekar BabuArtola, GarikoitzMarques, RuiArul, N. SabariMarques, CarlosArumugam, VeerakumarMarques, DuarteArvapally, Ravi K.Marquez, Francisco M.Aryan, PouriaMarquez, CarlosAsa'ad, FarahMárquez, AugustoAsadi, HamedMarradi, AlessandroAsadnia, MohsenMarrazza, GiovannaAsami, ToshihikoMarrazzo, PasqualeAseev, V. A.Marrocchi, AssuntaAsfin, Ruslan E.Marseglia, GuidoAsgharzadeh, AmirMarszalek, MartaAshok Kumar, MeiyazhaganMarta, Plonska-BrzezinskaAshton, TrentMartella, ChristianAskarinejad, SinaMartí Albiñana, José VicenteAslan, AhadiMartikka, OssiAslani, FarhadMartin, AidaAslanzadeh, SamiraMartin, Jose MiguelAšmontas, SteponasMartin, EricAssadi, AymenMartin, RichardAstakhov, Viktor P.Martincic, EmileAsteris, Panagiotis G.Martín-de León, JudithAstrakov, SergeyMartinelli, EnzoAta, SeisukeMartinelli, MichelaAtabaev, TimurMartinetti, AlbertoAtanase, LeonardMartinez, SanjaAthanasiou, ChristosMartínez, Borja ArroyoAtrens, AndrejMartínez-Calderon, M.Atroshchenko, ElenaMartinez-Cazares, Gabriela M.Atsawasuwan, PhimonMartínez-Espinosa, Rosa MaríaAtta-Obeng, EmmanuelMartínez-Lillo, JoséAttar, HooyarMartinez-Martinez, DiegoAttila, NagyMartínez-Martínez, Juan EnriqueAtuchin, Victor V.Martinez-Triguero, JoaquinAtwater, Harry A.Martinho, Fernando C. G.Auclair, NicolasMartini, CarlaAuffermann, GudrunMartini, AshlieAuger, Maria A.Martinka, JozefAugustyniak, AdamMartino, MarcoAversa, LucreziaMartino, SabataAvila-Paredes, Hugo J.Martin-Ramos, PabloAvilés, M.D.Martins, Andre F.Awana, V.P.SMartins, José A.Axinte, EugenMartins, PauloAyala, JuliaMartins, Marcos SilvaAyaso, Francisco-JavierMartins, Rui C.Ayat, NadiaMartins, Luísa MargaridaAyoub, AshrafMartins, RodrigoAytemiz, DeryaMartins Amaro, Ana Paula BetencourtAyukawa, YasunoriMarto, Carlos MiguelAyyagari, AdityaMartone, AlfonsoAyyavoo, JayalakshmiMartorana, AntoninoAzarbayejani, MohammadMartowicz, AdamAzimi, MohsenMartucci, AnnalisaAzina, ClioMartynenko, NataliaAzoug, AurelieMaruda, Radosław W.B. Rogov, AlekseyMarx, MichaelBaaße, AnnemarieMarycz, KrzysztofBaba, KoumeiMarzec, AnnaBaba, Nadim Z.Marzec, MichałBabaniaris, StevenMasaharu, NakayamaBabarao, RavichandarMasania, KunalBabich, LeonidMasashi, IshikawaBabo, PedroMasayuki, NiheiBabu, BathulaMascaretti, LucaBaca, Justin TMashifana, TebogoBacardit, AnnaMasi, GiuliaBaccarani, GiorgioMasłowski, MarcinBacciocchi, MicheleMassaro, MarinaBachagha, TarekMassey, Caleb P.Bacigalupo, AndreaMastali, MohammadBadawy, Amro ElMastalska-Popławska, JoannaBadea Doni, MihaelaMastanjević, KristinaBadilescu, SimonaMasuda, RyoBadogiannis, EfstratiosMatache, GheorgheBae, Sung-ChulMateos, AngelBae, Hyung D.Mateus, João MascarenhasBae, WoorhamMathijssen, Arnold J. T. M.Bae, DowonMathis, KristianBae, ChangdeuckMathivathanan, LogeshBae, Youn-SangMathur, SanjayBae, KihoMatiadis, DimitrisBae, Jae WungMatko, VojkoBae, HyungdaeMatos, InêsBaena, Francisco ManuelMatras, AndrzejBaffari, DarioMatsubara, MasahikoBagdziunas, GintautasMatsuda, MitsuhiroBaghdasaryan, TigranMatsui, JunBagherpour, EbadMatsushima, YutaBagio, VincenzoMatsushita, MasafumiBagnato, GiuseppeMatsuura, KazunoriBahador, AbdollahMattei, VincenzoBahk, Je-HyeongMatteis, Valeria DeBahmani, AramMatthews, DaveBai, HongweiMattioli, MicheleBai, MingfengMatula, GrzegorzBai, MingwenMatwijczuk, ArkadiuszBai, ChengyingMatykiewicz, DanutaBaimova, Julia A.Matys, JacekBaino, FrancescoMaurice, DavidBajare, DianaMauriello, FrancescoBajda, TomaszMauro, NicolòBaji, AvinashMauropoulos, AzariasBajpai, AnkurMavromatidis, LazarosBaker, MatthewMaximov, MaximBaker, PaulMayans, JuliaBakhshian, SaharMayencourt, PaulBakir, NasimMayer, ThomasBakowsky, UdoMayer-Gall, ThomasBakunowicz, JerzyMayuet-Ares, PedroBalachandran Nair, ShanoobMazalski, PiotrBalaguru, RajavelMazancová, JanaBalalaeva, Irina V.Mazari, MehranBalamurugan, JayaramanMaździarz, MarcinBalandin, Alexander A.Mazeika, LiudasBalart, MariaMazilu, ClaudiuBalashova, E. V.Mazumder, MithilBalasingam, Suresh KannanMazur, MartaBalasubramanian, KarthikMazur-Bialy, Agnieszka IrenaBalazsi, CsabaMazurek, GrzegorzBalcar, HynekMazzanti, ValentinaBalcerczyk, AnetaMazzocchetti, LauraBalda, RolindesMbey, Jean AimeBaldo, NicolaMcCrory, JohnBalevicius, ZigmasMcDonnell, Stephen J.Bálint, ErikaMcGee-Lawrence, MeghanBalis, NikolaosMcGilly, Leo J.Balle, FrankMcHugh, KevinBallesta-Claver, JulioMcIntyre, Dustin L.Balme, SébastienMcLean, AlexanderBalobanov, ViacheslavMcMaster, Scott A.Balonis, MagdalenaMcMillan, Alison J.Baltazar, Luís Gonçalo CorreiaMeaud, JulienBaltriukiene, DaivaMedel, Francisco JavierBanach, MarcinMederic, PascalBanan Sadeghian, RaminMederski, Piotr S.Banaszkiewicz, TomaszMedina Gallego, JuanBandala, Erick R.Medrano Jimenez, Jose A.Bandari, Yashwanth KumarMedunić, GordanaBandyopadhyay, SulalitMedvecky, LubomirBandyopadhyay, AmitMedved, JožefBanerjee, AmborishMedved, SergejBanerjee, Shib ShankarMedvedev, AndreyBanerjee, AbhinandanMedvedev, DmitryBanerjee, AnilMedvedev, DmitriBani, DanieleMedyński, DanielBankova, VassyaMeen, Teen-­HangBañón, FermínMeenashisundaram, Ganesh KumarBao, YinyinMegalini, LudovicoBao, ChunhuiMegiel, ElżbietaBao, YiMeher, SubhashishBao, JieMehrali, MehdiBarabas, SorinMehrpouya, MehrshadBarabás, RékaMei, HanfeiBaran, TomaszMei, BastianBaranowski, PawełMeijs, SuzanBárány, TamásMeille, ValerieBarbeck, MikeMelánová, KláraBarberis, AntonioMelchers, RobertBarberis, FabrizioMelnykowycz, MarkBarbi, SilviaMeloni, EugenioBarbieri, GiuseppeMelucci, DoraBarbosa, JoaquimMemon, Faisal AhmedBarboutis, Ioannis A.Memon, SaimBarbudo, AuxiMena, Vicente FernandoBarczewski, MateuszMenale, CiroBardi, GiuseppeMendecki, LukaszBarea, RafaelMendes, JoaoBarghabany, PeymanMendiguren, JosebaBaricco, MarcelloMendoza, Rodolfo JrBarile, ClaudiaMeneghetti, GiovanniBarille, RegisMeneguzzo, FrancescoBarisic, IvanaMenendez, MiguelBarkoula, Nektaria-MarianthiMenezes, PrashanthBarman, ManikMenezes, PradeepBarnat-Hunek, DanutaMeng, WeinaBaron, ChristianMeng, XinBaron, MarcoMeng, XiangchaoBarretta, RaffaeleMeng, QingshiBarris, CristinaMengucci, PaoloBarroso-Solares, SusetMenichetti, GuidoBartkowiak, TomaszMensik, MiroslavBartkowska, AnetaMenzel, StephanBartnicki, JarosławMenzler, NorbertBartocci, PietroMercer, JohnBartocha, DariuszMercone, SilvanaBartoli, MattiaMerie, Violeta ValentinaBartolomeo, Antonio DiMerkel, MarkusBartolotti, IsabellaMerodio, JoseBartoňová, LucieMeroni, DanielaBartuli, CeciliaMerrett, Craig G.Baruth, AndrewMertas, AnnaBasak, AmritaMertens, AnneBasaran, CemalMertens, ChristianBasiak, EwelinaMertz, DamienBaskoutas, SotiriosMesa, FranciscoBasoli, FrancescoMesarovic, SinisaBassini, EmilioMesquita, ConceiçãoBäßler, RalphMesquita-Guimarães, JoanaBassman, Lori C.Messa, Gianandrea VittorioBasso, VittorioMessyasz, BeataBastakoti, Bishnu PrasadMester, Patrick-JulianBastidas, David M.Mészàros, IstvànBastola, EbinMetson, James BernardBastos, JulioMetzinger, LaurentBasu-Gupta, SudeshnaMeyer, Jerry R.Batagelj, BostjanMia, MozammelBatail, PatrickMiao, YinghaoBatista, MoisesMiao, YuBatmunkh, MunkhbayarMiarka, PetrBattaglia, VincentMicci, MichaelBattaglino, Ricardo A.Micek-Ilnicka, A.Battistel, AlbertoMichail, ChristosBattistoni, SilviaMichalcová, AlenaBatuk, MariaMichalik, JanBatule, Bhagwan SahebraoMichaud, Jean-FrancoisBauer, WolfgangMichelini, ElenaBauer, FrancoisMicó-Vicent, BàrbaraBauer, A.J.Miculescu, FlorinBaumann, Felix W.Miculescu, MarianBaumeister, JoachimMicutz, MarinBaxavanis, TheocharisMie, YasuhiroBayer, IlkerMiele, ErmannoBayraktar, EminMieloszyk, MagdalenaBayu Aji, Leonardus BimoMierzejewska, Żaneta AnnaBazhenov, ViacheslavMierzwiński, DariuszBazrafshan, ZahraMigita, SatoshiBazzaz, MohammadMigliaccio, LudovicoBeal, ColineMiguel Eguía, ValentínBeard, James D.Miguel Oliveira, LuisBeata, ZimaMihai, MaraBeben, DamianMihelčič, MohorBecht, Jean-MichelMikhaylov, AlexeyBecker, KathrinMikhaylovskaya, AnastasiaBedia, JorgeMiki, HajimeBediz, BekirMiki, TakahitoBednarz, SzczepanMiklaszewski, AndrzejBedo, TiborMikolajczyk, TadeuszBedon, ChiaraMikucioniene, DaivaBeex, LarsMilanesio, MarcoBegaud, XavierMilani, GabrieleBegg, DavidMilavetz, BarryBeghetto, ValentinaMilčius, DariusBehnam, BehrouzMilcovich, GesmiBehnood, AliMilczewska, KasyldaBehúlová, MáriaMilenin, AndrijBeke, DezsoMiletić, Goran I.Bekmukhamedov, Giyjaz E.Milia, EgleBelavič, DarkoMilicevic, KrunoBełdowski, PiotrMilinovic, AndrijanaBeligni, AlessioMilionis, AthanasiosBelkin, Pavel N.Militzer, MatthiasBell, Carl-MartinMilkovič, OndrejBella, FedericoMilla, JoseBellantone, VincenzoMillithaler, Jean-FrançoisBellardita, MariannaMills, DavidBellini, CostanzoMilosan, IoanBellisario, DeniseMilovanovic, BojanBelloncle, ChristopheMiluski, PiotrBelousov, PetrMin, Kyeong-SikBelova, KseniaMin, Dong JoonBeltran, Ana M.Min, Byeong JuneBeltrán, HéctorMin, RuiBelzunce, Francisco JavierMin, Kyung-SanBen-Artzy, AdiMinak, GiangiacomoBenco, LubomirMinakshi, ManickamBende, AttilaMinami, IchiroBender, MarkusMinami, YasunoriBender, PhilippMinamiki, TsukuruBender, FlorianMiñano, IsabelBenedetti, AlessandroMinařík, AntonínBenedetti, MatteoMinato, Ken-ichiroBenesperi, IacopoMincheva, RosicaBenetti, MassimilianoMindaugas, GedvilasBeniah, GoliathMindemark, JonasBenoit, DenizotMine, YuichiBenseman, Timothy M.Minea, Alina AdrianaBera, GopalMinguez, RikardoBeranoagirre, AitorMínguez-Algarra, J.Berardino, Santino Eugénio DiMinguzzi, AlessandroBerbecaru, Andrei ConstantinMiniaci, MarcoBéreaux, YvesMino, LorenzoBerenjian, AydinMinohara, MakotoBerge, FranzMiranda, João M.Berillis, PanagiotisMiritello, MariaBerisha, BekimMirkhani, Seyyed AlirezaBerkson, ZachariahMiron, Richard J.Berlowska, JoannaMiroshnichenko, AndreyBernardeta, DębskaMiroslaw, WesolowskiBernardi, SaraMirsayar, MirMiladBernardini, SandrineMirski, RadosławBernardo, LuísMirzaali, Mohammad J.Bernardo, PaolaMirzaei, AliBernardo, GabrielMishra, KunalBernards, MatthewMishra, RajeshBernechea, MaríaMishra, Pawan KumarBernède, Jean ChristianMishra, Rupesh K.Bernhard, HofkoMitani, AtsushiBerski, WiktorMitchell, Scott G.Berta, Gómez-IorMitoraj-Królikowska, MarzenaBertani, RobertaMitran, GheorghiţaBerteau, Jean-PhilippeMitrica, DumitruBerthe, JulienMitryukovskiy, SergeyBertini, LeonardoMittal, NiteshBerto, MarcelloMittova, Irina YaBertoglio, FedericoMiu, DanaBertolotti, FedericaMiyaji, HirofumiBertsch, ArnaudMiyamori, YasunoriBesagni, GiorgioMiyamoto, KatsuhikoBeskopylny, AlexeyMiyamoto, HiroyukiBesnard, AurélienMiyawaki, RitsuroBespalko, Yulia N.Miyazaki, ToshikiBessmertnykh-Lemeune, AllaMizera, AlesBetke, UlfMizzi, LukeBettache, NadirMłynarczykowska, AnnaBettini, SimonaMoad, GraemeBhagia, SamarthyaMobed-Miremadi, MaryamBhandari, KhagendraMobili, AlessandraBhat, SartajMoccia, MassimoBhattacharya, ArunodayaModica, MariaBhattacharya, SumitMoeini, GhazalBhattarai, Jay K.Mogeritsch, JohannBhowmick, SukantaMoghaddam, Hesam S.Bhuiyan, YeasinMohajerani, AbbasBiałobrzeska, BeataMohamed Ali, Mohamed SadakkathullaBiałowiec, AndrzejMohamedali, KhalidBidulský, RóbertMohammadi, FazelBieda, BogusławMohammadiarani, HosseinBielejewski, MichałMohammed, AlyaaBienias, JaroslawMohan, Velram BalajiBieniek, TomaszMohanta, AntaryamiBiernasiuk, AnnaMohd-Yasin, FaisalBiernat, JanMohles, VolkerBiesuz, MattiaMohn, DirkBihamta, RezaMohtadi Bonab, Mohammad AliBikiaris, DimitriosMokaya, RobertBílek, VlastimilMoldovan, HoraţiuBilenberg, BrianMolina, C.B.Billingham, JohnMolinari, AlbertoBini, MarcellaMoliner, CristinaBiondi, LorenaMolla, Md. Ashraful Islam MollaBirch, CatherineMollenhauer, KonradBirleanu, CorinaMolnar, ViktorBirowska, MagdalenaMolnarova, OrsolyaBisbey, RyanMombelli, DavideBiscaia, Hugo C.Monai, MatteoBischof, SandraMonchau, FrancineBisoyi, Hari KrishnaMondal, SudipBiswas, PranabMondal, BikashBisz, ElwiraMonetta, TullioBizzarri, ClaudiaMonfort, OlivierBjorklund, RobertMonguzzi, AngeloBjörkman, TorbjörnMonkova, KatarinaBjörling, MarcusMonny, Sabiha AkterBlack, LeonMonopoli, MarcoBlack, LachlanMonroe, Mary Beth BrowningBlaga, LucianMonsalve, AlbertoBlanche, Pierre-alexandreMontagne, KevinBlanco, AngelesMontalto, LuigiBlanco, IgnazioMontanari, RobertoBlanco, DavidMontealegre-Melendez, IsabelBlaschke, Daniel N.Monteiro, Ricardo Nuno CarvalhoBlázquez Gámez, Javier S.Montero, XabierBleackley, Mark R.Montero, Rocío SánchezBłoch, KatarzynaMontero Sistiaga, Maria LuzBloise, NoraMontero-Chacón, FranciscoBlom, JohanMontiel-Company, José MaríaBloodgood, Matthew A.Montuori, RosarioBlum, WolfgangMonzón, AntonioBobadilla, Luis F.Monzón, Mario D.Bobinger, MarcoMoon, Hong ChulBobruk, Elena VladimirovnaMoon, JuhyukBoccarusso, LucaMoon, Young HoonBochenek, DariuszMoon, Seung-JaeBocîrnea, Amelia ElenaMoon, KihoonBoda, Sunil KumarMorales-Conde, Maria JesúsBoddaert, XavierMorales-Narváez, EdenBoddapati, LoukyaMorales-Rivas, LuciaBoddeti, NarasimhaMorales-Rodríguez, AnaBodini, MatteoMorales-Vidal, MartaBogacki, JanMoramarco, VincenzoBogdanowicz, KrzysztofMoratti, StephenBöhm, MichałMoravcik, IgorBoillot, Marie LaureMorazzoni, FrancaBoinovich, Ludmila B.Moreau, JulienBolander, JohnMoreirinha, CatarinaBolelli, GiovanniMoreno, JovitaBollinger, Jean-ClaudeMoreno Pineda, EufemioBolotov, LeonidMoreno-Maroto, José ManuelBolz, SebastianMoreno-Piraján, Juan CarlosBombac, DavidMoretti, LauraBompa, DanMori, KotaroBompos, DimitriosMori, SeijiBon, VolodymyrMorikawa, AtsushiBonek, Mirosław S.Morini, MirkoBonifacio, Maria AddolorataMoritz, MichałBöning, KlausMorley, Nicola ABonomo, MatteoMoroni, DavideBontempi, ElzaMoroz, AdamBonyár, AttilaMorozova, SofiaBorcia, GabrielaMorreale, MarcoBordeenithikasem, PunnathatMorris, GordonBorek, WojciechMorrison, Gregory M.Borela, RodrigoMorrison, FinlayBorge Diez, DavidMortimer, MonikaBorges, JoelMortula, MarufBorgesen, PeterMorvan, BrunoBorghetti, MichelaMosa, IslamBorgioli, FrancescaMoscone, DanilaBoriani, StefanoMoseke, ClausBoris, RenataMosk, AllardBorisenko, Elena B.Mosleh, Ahmed O.Borkowski, LeszekMostafa, AhmadBorla, OscarMostovshchikov, Andrei V.Bormashenko, EdwardMott, Derrick M.Borodachenkova, MarinaMöttönen, VeikkoBorodianskiy, KonstantinMotyka, MaciejBorodin, ElijahMotzki, PaulBorowicz, MarcinMoulick, AmitavaBorowiec, JoannaMouralova, KaterinaBortot, ElianaMourão, Carlos FernandoBorůvka, VlastimilMourdikoudis, StefanosBorz, Stelian AlexandruMoury, RomainBorzabadi-Farahani, AliMouzakis, Harris P.Bos, FreekMoya, JoséS SerafínBoshkov, NikolaiMozaffari, SaeedBosnjak, JosipaMozetic, MiranBotelho, MonicaMráček, AlešBothe, Kyle M.Mrázek, JanBotto, DanieleMrlík, MiroslavBou, Santiago FerrandizMroczyński, RobertBouazizi, NabilMrówczyńska, MariaBouby, CélineMrowczynski, RadoslawBoumerzoug, ZakariaMróz, WaldemarBourebrab, MarionMu, Q.X.Bouropoulos, NikolaosMucha, JacekBoutami, SalimMück, MichaelBouville, FlorianMudunuru, Maruti KumarBoyard, NicolasMuhammad Kashif.Boyd, AnthonyMukai, KeisukeBozorgzad, AshkanMukherjee, TuhinBraem, AnnabelMukherji, DebashisBraga, Daniel F.O.Mulholland, TomBranco, JorgeMüller, GerhardBrand, Alexander S.Müller, AnetteBranda, FrancescoMüller, KarstenBrandus, Catalina-AliceMüller, MichaelBranicio, PauloMulton, StéphaneBrantley, William A.Münch, AlexanderBranzei, MihaiMunirathinam, RajeshBranzoi, FlorinaMunjiza, AnteBrassard, Jean-DenisMuñoz, ÁngelBrazdil, James F.Muñoz, MartaBrcic, MarinoMuñoz, Alexander NavarreteBreakah, TamerMuñoz-Sánchez, BelénBreaz, Radu EugenMunteanu, Florentina-DanielaBrechtl, JamiesonMünzenrieder, NikoBreidenstein, BerndMurariu, Alin ConstantinBreitbarth, EricMurčinková, ZuzanaBrela, MateuszMurillo-Marrodán, AlbertoBrendel, LotharMuro, MaiderBreslin, Carmel B.Murshid, NimerBrestic, MarianMurthy, T. S. R. ChBringuier, StefanMurugan, N. ArulBroda, JanMusić, SvetozarBrosed, Francisco JavierMusioł, MartaBrosseau, ChristianMusmarra, DinoBrostow, WitoldMusselman, EricBrouwers, JosMusso, SimoneBrowar, Andrew W.Musteata, MihaiBru, KathyMusumeci, GiuseppeBruckner, GudrunMuszka, KrzysztofBruckner, ChristianMusztyfaga-Staszuk, MałgorzataBrucoli, MatteoMuszyński, SiemowitBrudnyi, ValentinMutch, GregBrühwiler, DominikMuthuraj, RajendranBrunčko, MihaelMuydinov, RuslanBrunesi, EmanueleMuziol, Tadeusz MikolajBruni, CarloMuzyk, MarekBrutti, SergioMyers, OliverBrychcy, EwaMyers, Damian E.Bryja, Leszek BryjaMykhalenkov, KostainatynBrzeska, JoannaMyllymaa, SamiBrzózka, AgnieszkaMynbaeva, MarinaBrzyski, PrzemysławMystkowska, JoannaBuaki-Sogó, MireiaNa, SeungukBubnov, AlexeyNabae, YutaBuchberger, AntonNabeel, MuhammadBuchely, MarioNadolny, KrzysztofBuchet, ReneNadolny, ZbigniewBuchmayr, BrunoNaftaly, MiraBucinskas, VytautasNagai, MasayaBuckner, Steven W.Nagai, KoheiBuczyński, PrzemysławNagai, HirokiBudhi, SridharNagao, DaisukeBudnyak, TetyanaNagaraj, ChandranBuerger, IngaNagasawa, TsuyoshiBugaev, AramNagel, JuergenBugajski, PiotrNaghilou, AidaBugajski, MaciejNagit, GheorgheBuhl, JohannesNagumo, MichihikoBui, Tinh QuocNagy, BalázsBui, Ngoc KienNagy, EndreBuj Corral, IreneNahata, SudhanshuBulgariu, LauraNaik, ShivangiBulic, MladenNair, Sriramya D.Bulzak, TomaszNair, Sandeep SudhakaranBumgardner, Joel D.Najdahmadi, AvidBureš, RadovanNajlah, MohammadBurger, Michael C.Najm, Husam S.Burja, JakaNakashima, SatoruBurkle, DannyNakazawa, HidekiBürklein, SebastianNakhli Mahal, HoumanBurlacu, AlexandrinaNakielski, PawelBurpo, Fred J.Naleway, StevenBurrascano, PietroNam, TranVanBursi, LucaNam, JeongsooBusby, YanNam, Seung YunBusquets, Maria AntòniaNama, NiteshBustillo, JulienNambu, KoichiroButicchi, GiampaoloNamburi, Devendra KumarButrymowicz, DariuszNameni, GordonButt, JavaidNanaki, StavroulaBystrov, VladimirNand, AshveenBzówka, JoannaNanda, Sitansu SekharCabaj, JoannaNanev, ChristoCabanelas, Juan CarlosNano, AdelaCabboi, AlessandroNanver, Lis K.Cabello-Alvarado, Christian J.Napa, Sae-BaeCabeza Simo, MartaNaplocha, KrzysztofCabrales, LuisNarayanan, Remya P.Cabrera, Manuel MontenegroNarayanan Nair, MayaCabrera-Codony, AlbaNarciso, JavierCaccia, MarioNardecchia, FabioCaesarendra, WahyuNariman, EnikeevCaggiano, AntonioNarloch, PiotrCahill, JamesNasir, OthmanCai, FengNasir, JamalCai, QizhouNäslund, Lars-ÅkeCai, JinguangNassar, HusseinCaiazzo, FabriziaNastase, FlorinCalà Lesina, AntoninoNastuta, Andrei VasileCalabrese, LuigiNatalello, AntoninoCalabrò, Paolo S.Natali Sora, IsabellaCalaf Chica, JoseNataraj, LathaCalahorra, YonatanNatsui, ShungoCalamita, GiuseppeNattestad, AndrewCalandra, PietroNaumova, Ella A.Caldas, PauloNavabpour, ParniaCalero, Olga CaballeroNavarro, RodrigoCaliendo, CinziaNavarro, Francisco JavierCalvaresi, MatteoNavas, JavierCalvillo, LauraNavau, CarlesCalvino-Gámez, Jose JuanNavin, Chelliah V.Calvo Guirado, José LuisNazare, ShonaliCalvo-Dahlborg, MoniqueNazarian-Samani, MasoudCalvo-Guirado, José LuisNazarov, Andrej P.Calzaferri, GionNazarov, Denis V.Camblor, Miguel A.Nazir, TariqCambronero, Luis Enrique G.Nazir, HammadCamelia, GaborNdieyira, JosephCamerlingo, CarloNeagu, AdrianCameron, JosephNeal, LukeCameron, DavidNeamt, LiviuCametti, CesareNečasová, BarboraCaminero Torija, Miguel ÁngelNedeljkovic, MarijaCamões, AiresNee, Matthew J.Campbell, JohnNeel, Ensanya A. AbouCampbell, KrisNegahban, MehrdadCampiglio, Chiara EmmaNegahdar, LeilaCampillo, Jose MiguelNegulescu, IoanCañavate, JavierNejadi, ShamiCancellieri, ClaudiaNemes, Norbert M.Candau, NicolasNemes, CiprianCandolfi, ChristopheNemeş, OvidiuCanetta, ElisabettaNémeth, RobertCangialosi, DanieleNepomuceno, MiguelCaniato, MarcoNeslušan, MiroslavCannilla, CatiaNesterenko, Vitali F.Cannone Falchetto, AugustoNesterov, DmytroCanteli, DavidNetskina, OlgaCantera, M. AsunNeuenschwander, JuergCantisani, GiuseppeNeufurth, MeikCao, Hoang-LongNeves, José Manuel CoelhoCao, BoNeves, RuiCapasso, RaffaeleNeves, FilipeCapitão, Silvino DiasNeves, Cristina Sofia Dos SantosCaplins, Benjamin W.Nevshupa, Roman A.Čaplovič, ĽubomírNezafati, MarjanCapodaglio, Andrea G.Ng, P.L.Caprara, SergioNg, Tang-TatCapsoni, DorettaNg, Alex Ching TaiCapurso, GiovanniNg, Wei LongCaputi, Lorenzo S.Ngamcherdtrakul, WorapolCaputo, PaolinoNganga, SaraCarabineiro, SóniaNgene, PeterCaravelli, FrancescoNgo, Duc-TheCarballo, JavierNgo, Chi-VinhCarbas, R.J.C.Nguyen, Nhat TruongCarbone, LorenzoNguyen, Van BacCarbone, ClaudiaNguyen, Sy-NgocCárcel-Carrrasco, Francisco JavierNguyen, Thanh-TungCárdenas, SoledadNguyen, Van KhanhCardenas-Morcoso, DrialysNguyen, TrieuCardiff, PhilipNi, DalongCardinale, AnnaNian, QiongCardon, LudwigNicholls, Luke H.Cardoso, DavidNicholson, John W.Carey, BenjaminNicoara, MirceaCarli, RaffaeleNicodemo, GianfrancoCarlucci, ClaudiaNicola, LolliCarmen, Martínez GarcíaNicolai, JulienCarneiro, José RicardoNicolas, AndreaCarou, DiegoNicoletta, Fiore PasqualeCarraro, FrancescoNicolini, AndreaCarrilho, EuniceNiculescu, VioletaCarsí, MartaNie, YiCarty, William M.Niemczewska-Wojcik, MagdalenaCarvalho, Luisa M. H.Niemczyk, AgataCasagrande, AngeloNieminen, PenttiCasalino, GiuseppeNiemirowicz-Laskowska, KatarzynaCasarejos, EnriqueNiesłony, PiotrCasassa, SilviaNieto, DanielCascardi, AlessioNieto, AndyCase, RaymundoNiewa, RainerCasey, SeanNikishin, SergeyCasimiro, Maria HelenaNikitas, NikolaosCassetta, MicheleNikolaidis, Alexandros K.Castaldo, PaoloNikolic, DraganCastanheira, Elisabete M.S.Nikumb, SuwasCastaño, OscarNilimaa, JonnyCastedo, RicardoNilsson, FritjofCastell, PereNimbalkar, SanjayCastellano, Nuria NovasNing, JinqiangCastellano, CarloNishida, TakahiroCastelli, Ivano E.Nishikiori, HiromasaCastellini, ElenaNishinaka, HiroyukiCastellino, MicaelaNishiumura, TomoyoshiCastilla, Ana M.Nishiwaki, TomoyaCastro, AndreNishiyabu, RyuheiCastro, EdisonNishiyama, NorimasaCastro Cerda, Felipe M.Nisnevitch, MarinaCastro-Colin, MiguelNistor, Ileana DenisaCastro-Fresno, DanielNitschke, MirkoCastro-Gomes, JoãoNitta, Koh-heiCataldo, AntoninoNiu, YulingCatizzone, EnricoNiu, ChangningCattaneo, SaraNiv, AviCattoën, XavierNiwano, MichioCauwe, MaartenNjock Bayock, FrancoisCavallaro, GiuseppeNobile, LucioCavallo, DarioNoce, CanioCazacu, EmilNocentini, SaraCecchel, SilviaNocivin, AnnaCecconet, DanieleNoel, MartinCech, VladimirNoga, MarcinCecilia, Juan AntonioNohe, AnjaCecone, ClaudioNokken, MichelleČekon, MiroslavNomura, NaoyukiCelano, UmbertoNoor E Khuda, SarkarCelauro, ClaraNoorani, Rafiqul I.Celentano, MaurizioNoordergraaf, Inge-WillemCelentano, DiegoNord, NatasaCelikbilek, OzdenNordseth, ØrnulfCelzard, AlainNorek, MałgorzataCengiz, SezginNoréus, DagCerbu, CameliaNorouzzadeh, PayamCereceda, DavidNorton, MichaelCeresoli, DavideNosko, OleksiiCeretti, MonicaNosoudi, NasimCerjan, BenjaminNouranian, SasanCernescu, AnghelNovais, Rui Miguel TeixeiraCerny, MiroslavNovák, PavelCerrada, Maria LuisaNóvoa, X. RamónCerrone, FedericoNowak, WojciechCerroni, BarbaraNowak, AdrianaCerro-Prada, ElenaNowak, DamianCervino, GabrieleNowak-Michta, AnetaCesano, FedericoNowicki, MichałCesarz-Andraczke, KatarzynaNowicki, JanuszCha, Sung WoonNowoświat, ArturCha, Sang-RyulNsugbe, EjayChae, MichaelNtougias, SpyridonChakrabarti, AmartyaNuckowski, PawełChakravarty, ShatadruNuić, IvonaChaliampalias, DimitriosNunes, MartaChalioris, ConstantinNunes, DanielaChamaani, AmirNunes, Carla D.Chami, BelalNunes, LeonelChandrasekaran, MurugesanNuñez, NeftaliChang, Yao-FengNunna, Bharath BabuChang, Yuan-JenNypelo, TiinaChang, Jui-ChengOancea, GheorgheChang, Wei-JenObataya, EiichiChang, Sheng-PoObonyo, EstherChang, MinwooObraztsov, Alexander N.Chang, Dong WookOchi, MasayukiChang, Wen-ShaoOchiai, TsuyoshiChang, S.Y.Ochowiak, MarekChang, Tsui-LingOda, TakujiChangzeng, YanOdintsova, GalinaChantana, JakapanOesch, TylerChao, ZhouOestreicher, VíctorChao, HuikuanOffermanns, VincentChao, Chia-TerO'flaherty, FinCharisiou, NikolaosOgale, Amod A.Charles, Andrew D. M.Ogam, ErickCharmas, BarbaraOgawa, KeijiCharvatova, HanaOgawa, YukikoChatare, VijayOgawa, ToshioChatterjee, SabornieOgawa, ShinpeiChatterjee, SudiptaOgierman, WitoldChaturvedi, PracheeOgino, IsaoChatzichristidi, MargaritaOgirigbo, Okiemute RolandChaudhari, SmrutiOh, HyunchulChauhan, GarimaOh, TaekeunChauhan, NeerajOh, Sang-KeunChava, Rama KrishnaOh, Seung-HwanChavan, Sachin MarutiOh, TeresaChaves, IgorOh, HongseobChavez-Angel, EmigdioOhashi, MasashiChavoshi, Saeed ZareOhlckers, PerChawake, NirajOhno, SeigoChawla, NehaOhno, NobutadaCheca, SaraOhsawa, TakeoCheetham, PeterOhsedo, YutakaChehimi, MohamedOhta, HiromichiChehroudi, BabakOhtani, NoboruCheikh, Khadija ElOikawa, KatsunariChekalkin, TimofeyOjovan, MichaelChekurov, SergeiOk, Myoung-RyulChen, Tao-HsingOkamoto, MasamiChen, CanOkayasu, MitsuhiroChen, JunOkazaki, JojiChen, ZhongbingOkhay, Olena I.Chen, YueOkitsu, YoshitakaChen, Hui-SuOklobia, OchaiChen, Ruei-TangOkpin, NaChen, ZhitongOkrajni, JerzyChen, Kuo-PingOksiuta, ZbigniewChen, FangOkubo, MasaakiChen, MingshengOkumura, SusumuChen, HouyangOkunkova, Anna A.Chen, HuizhiOkyay, GizemChen, XinOladapo, BankoleChen, Tai-ChengOlea, MariaChen, Wei-ChunOledzka, EwaChen, Teng-MingOlejnik, KonradChen, Roland K.Olejníková, PetraChen, ShuoOlewnik-Kruszkowska, EwaChen, FengOliker, VladimirChen, JiahaoOliveira, João PedroChen, Jian-ZhangOliveira, GustavoChen, ChunhuiOliveira, MiguelChen, I-ChenOliveira, Joel R.M.Chen, SherryOliveira, MonicaChen, XiyuanOliveira, Serafim M.Chen, ChiachungOliver, RachelChen, WenwuOliveri, Roberto LuigiChen, ChuantongOliver-Ortega, HelenaChen, DaiqinOliynyk, Anton O.Chen, T. H.Olofinjana, AyodeleChen, WeishengOlsson, MikaelChen, Kuo-YuOlvera-Vargas, HugoChen, YijiaOmar, AhmedChen, Hone-ZernOmbres, LucianoChen, Hsuan-YingOmrani, EmadChen, Yi-WenOms, OlivierChenchev, IvanOnaka, SusumuCheng, JiangOndra, VaclavCheng, Hsu-HuiOnisor, FlorinCheng, Chih-ChiaOno, KanjiCheng, ZhiqiangOono, NaokoCheng, GuangmingOpra, DenisCheng, PeiOprea, Corneliu IoanCheng, ZhenzhouOptasanu, VirgilCheng, Yang-TseOrazi, LeonardoCheng, ChengOrbulov, ImreCheng, XuanOrdonez-Miranda, JoseCheng, Chiang-HoOreshonkov, AleksandrChereddy, KiranOrlik-Kożdoń, BożenaCherng, Jyh-ShiarnOrlov, Alexei O.Chernyak, SergeiOrlov, V. M.Chernyshov, MikhailOrmeci, AlimChesnokov, AlexanderOrmellese, MarcoChetwynd, AndrewOrmondroyd, Graham A.Cheung, OceanOrtega, José MarcosCheung, Angus K.F.Ortega, JavierChiadighikaobi, Paschal ChimeremezeOrtega, ZaidaChiang, Ming-HsiOrtenzi, Marco A.Chiashi, ShoheiOsella, SilvioChiavaioli, FrancescoOsiński, PiotrChicardi, ErnestoOsipov, AndreyChicos, Lucia-AntonetaOsman Ahmed, AhmedChiesa, EnricaOsmanlic, FuadChiessi, EsterOsório, Jonas H.Chin, Cheng SiongOßwald, KaiChinh, Nguyen QuangOstapchuk, TetyanaChiniforush, Alireza A.Ostapiuk, MonikaChiolerio, AlessandroOstrowski, KrzysztofChirita, StanOsypenko, ArtemChirumamilla, ManoharOterkus, ErkanChiu, Chih-WeiOtmačić Ćurković, HelenaChiu, Te-WeiO'Toole, SaoirseChiu, ChunOtsuki, AkiraChizhik, Alexey I.Ottonelli, MassimoChladek, GrzegorzOuerdane, HenniChmielarz, PawełOueslati, WalidChmielewski, TomaszOuyang, LiangqiChmielewski, AndrzejOvchinnikov, AlexanderCho, ChungyeonOwen, RobertCho, LawrenceOwen, JoshuaCho, Jung SangOwolabi, GbadeboCho, Jong-RaeOyedele, AkinolaCho, Dae-HyunOzaki, TsuneyukiCho, Young-RaeOzbakkaloglu, TogayCho, SunghunOzgowicz, WojciechCho, JinhanPacaiova, HanaCho, Hyeon-YeolPacella, ManuelaChocyk, DariuszPacheco-Torgal, F.Choe, GyeongcheolPaci, MaurizioChoi, Yong GyuPaciorek-Sadowska, JoannaChoi, HeejooPadamati, Ramesh BabuChoi, Jane RuPadial-Molina, MiguelChoi, Soon MoPadil, Vinod V. T.Choi, Sung WoongPadmanabhan, JagannathChoi, Myong YongPaegle, IevaChoi, SeongHoPaeglitis, AinarsChoi, SejinPaek, Yeong-KyeunChoi, HeechaePagani, StefaniaChoi, Nag JungPagano, StefanoChoi, WonjaePage, JonathanChoi, Kyoung-KyuPahinkar, Darshan G.Choi, HeesupPahlevani, FarshidChoi, HeekyuPaidar, VaclavChokkalingam, AnandPaiè, PetraChomicz-Kowalska, AnnaPaik, HanjongChong, William W.F.Paillard, CharlesChorazy, SzymonPaipetis, AlkiviadisChortos, AlexPająk, MałgorzataChorzepa, Mi G.Pakdel, EsfandiarChotěborský, RostislavPakrashi, VikramChoubar, Mohammad AkbariPal, NabamitaChowdhury, PiyasPal, AnirbanChráska, TomášPalacz, MagdalenaChremos, AlexandrosPalamà, IlariaChrissafis, KonstantinosPalcut, MariánChristova, DarinkaPalermo, GiovannaChronopoulos, DimitriosPaleu, ViorelChronopoulou, LauraPalevicius, ArvydasChruściel, Jerzy J.Pálfalvi, LászlóChrysanthakopoulos, Nikolaos A.Palinko, IstvanChrysanthou, AndreasPalka, NorbertChrzan, Krystian LeonardPalma, Paulo J.Chua, BeeleePalma, PauloChuang, ChiashainPalma, LucaChuang, AndrewPalmero, PaolaChul Wee, LeePalmieri, MarcoChulkova, Tatiana G.Palomares, Antonio EduardoChun, JunhoPalou, MartinChundak, MykhailoPaluch, Andrew S.Chung, Sang-YeopPalumbo, DavideChung, Yong SukPalumbo, GaetanoChung, Ren-JeiPalumbo, CarlaChung, Sheng-HengPalumbo, OrieleChung, Shin HyePamies, RamonChuryumov, Alexander YuPan, ZhuChvála, AlešPan, YamingCiancaleoni, GianlucaPan, QinCicala, GianlucaPan, YeCicchi, StefanoPan, Hung-ChuanCicciù, MarcoPan, HaihuaCiccone, MarcoPanagopoulos, C. N.Cicconi, PaoloPanchenko, E. Yu.Cichomski, MichalPancielejko, MieczysławCichos, JakubPanda, BiranchiCiesielczyk, FilipPandele, MadalinaCiesielski, MichaelPandey, SudipCieslak, JakubPandey, GauravCieslar, MiroslavPandey, TribhuwanCifuentes, HectorPandiev, IvailoCinefra, MariaPanek, PiotrCinelli, PatriziaPánek, DavidCinteza, Otilia LudmilaPanich, Alexander M.Ciolaciu, DianaPanier, StephaneCiolacu, DianaPanikkanvalappil, Sajanlal R.Ciria, MiguelPanin, SergeyCiriello, ValentinaPanjan, PeterCirlugea, MihaelaPankratov, Evgeny L.Cirujano, FranciscoPanomsuwan, GasiditCiuffini, Andrea FrancescoPant, BishweshwarČižmár, ErikPantazopoulos, GeorgeČizmić, MirtaPantazopoulou, StavroulaClauss, MartinPantoya, MichelleClayton, John D.Panzarasa, GuidoClementi, FrancescoPaolini, RiccardoCoakley, EoinPaolini, Antonio EvangelistaCochis, AndreaPaolone, AnnalisaCochrane, CédricPap, JózsefCodetta-Raiteri, DanielePapa, ElettraCodispoti, RosamariaPapaccio, GianpaoloCodrean, CosminPapadopoulos, Antonios N.Coduri, MauroPapadopoulou, AlexandraCoelho, Leonardo BertolucciPapaefthymiou, SpyrosCoffetti, DennyPapageorgiou, Spyridon N.Coffinier, YannickPapalou, AngelikiCohen, YachinPapantonopoulos, GeorgeCojocaru, CorneliuPapatzani, StylianiCojocaru, VasilePapavasiliou, JoanCojocaru, AncaPapi, MassimilianoColangelo, FrancescoPapia, EvaggeliaColas, GuillaumePapon, Easir ArafatColeman, NicholaPapoulis, DimitrisColli, Alejandro NicolásPappas, George S.Collini, LucaPaprocka, IwonaCollins, SunnivaParadiso, PatriziaColniță, AliaParajuli, DurgaColom, XavierParakhonskiy, BogdanColomban, PhilippeParashchuk, TarasColombani, JeanPardanaud, CédricColombi, PierluigiParedes-Madrid, LeonelColosi, Horațiu AlexandruParigger, ChristianComan, CristinaParisi, FilippoCombescure, AlainPark, ChulhongComesaña, RafaelPark, SolmoiCometa, StefaniaPark, KwangsukConceição, JaimePark, Jun-BeomConcepción, PatriciaPark, Lee SoonConcilio, AntonioPark, JoontaekConcu, GiovannaPark, Joon SikConde, OlgaPark, Soo-JinConesa, José CarlosPark, Moon JipConnolly, James P.Park, Hyung-HoConradi, MarjetkaPark, JuhyunConstantinescu, HoriaPark, MiraConte, Antonietta LoPark, Chan HoConterosito, EleonoraPark, Min-SikContessi Negrini, NicolaPark, SangmoonConti, MassimoPark, JunyongContreras-Cáceres, RafaelPark, EnochCooke, KavianPark, Byung-WookCoppola, LuigiPark, Ji-ManCoppola, BartolomeoPark, Ji-WonCordaro, LuigiPark, JeyoungCordeiro, NereidaPark, ChanyeopCordero, FrancescoPark, Jay HoonĆorić, DankoParker, Stewart F.Corigliano, PasqualinoParola, StephaneCornu, DavidParth, PanchmatiaCoronado, Juan M.Parthasarathy, AnutthamanCorrea, Cinthia AntunesPartovi Nia, RachelCorreia, LuísParvez, KhaledCorreia, José A.F.O.Pasán, JorgeCorreia, Sandra F. H.Pascall, Andrew J.Cortese, LucaPascoe, John-AlanCortese, BarbaraPascual, LauraCosco, DonatoPascuta, PetruCosta, FrancoPasetto, MarcoCosta, Manuel Filipe P. C. M.Pashkin, OleksiyCosta-Almeida, RaquelPasinszki, TiborCostanzo, VincenzoPaskaleva, AlbenaCota, IulianaPasqua, LuigiCotet, CosminPasquini, EmilianoCoulter, Jonathan A.Passalis, NikolaosCoutinho, Paulo José GomesPassian, AliCoutinho, PaulaPasta, SalvatoreCox, SophiePašti, Igor A.Coy, EmersonPastor, José L.Craciun, Eduard-MariusPastore, RobertoCran, Marlene J.Pastore, TommasoCrans, DebbiePastorek, FilipCredico, Barbara DiPaszkiewicz, SandraCrespillo, Miguel L.Patanè, SalvatoreCretu, SpiridonPatel, HardikkumarCrisci, AlessandroPatel, VipulCristache, Corina MarilenaPater, ZbigniewCristea, DanPaternoster, CarloCristelo, Nuno MiguelPathak, PawanCristian, PetcuPathakoti, KavithaCristina, De NardiPatil, Swati J.Croatt, Mitchell P.Patil, ShradhaCrocombe, LeonardPatil, SachinCroitoru, CatalinPatini, RomeoCroll, Andrew B.Patra, SubirCrosky, AlanPatra, Hirak K.Crosnier, OlivierPatra, NiranjanCross, RodPattanaik, HimansuCross, Stephen A.Patterson, JenniferCrossley, AlisonPatterson, AlbertCrotti, ElenaPatterson, Albert E.Crucho, JoãoPattini, FrancescoCrucho, CarinaPaul, Suvash ChandraCruz, Rui M.S.Paul, BiplabCruz, SandraPaul, SanjoyCsaki, IoanaPaul Borg, RubenCsapo, EditPaula, Anabela BaptistaCsetenyi, Laszlo J.Paulin, IrenaCsík, AttilaPaulish, Andrey GeorgievichCsoka, LeventePaulo, CarlosCtibor, PavelPavaloaia, Vasile-DanielCuberes, TeresaPavić, LukaCudziło, StanisławPavlík, ZbyšekCuenca, EstefaniaPavlos, KlepetsanisCuesta, IvanPavlovic, AnaCuevas, FranciscoPavlů, TerezaCui, HaitaoPawar, RadheshyamCui, HaoPawar, ShitalCui, HaissiPawlak, MichałCulebras, MarioPawlak, WojciechCulshaw, BrianPawlak, RyszardCumming, Adam S.Pawlik, MarzenaCunnane, DanielPawlus, PawełCure, JérémyPazdera, LubošĆurković, LidijaPazniak, AnnaCurtis, Anthony D.M.Peale, RobertĆwiek, JanuszPeana, Massimiliano F.Cycoń, MariuszPecqueur, SébastienCygan, TomaszPedersen, HenrikCyganowski, PiotrPederson, RobertCyran, KatarzynaPedreño Rojas, Manuel AlejandroCzaja, PawełPedrini, GiancarloCzapik, PrzemysławPedzich, ZbigniewCzapik, AgnieszkaPeer, AkshitCzarnecka-Komorowska, DorotaPeer, PetraCzarnecki, Slawomir A.Peeters, RoosCzarnecki, LechPegoretti, AlessandroCzech, BożenaPei, ZingwayCzekanski, AleksanderPei, YanzhongCzeppe, TomaszPejkowski, ŁukaszCzerwinski, FrankPekin, Thomas C.Czigany, ZsoltPelegov, DmitryCzlonka, SylwiaPelin, Irina M.Czujko, TomaszPeljo, PekkaCzupryński, ArturPelka, RafałCzyzycki, MateuszPellegrini, GaiaD' Acunto, MarioPellejero, IsmaelD’Addazio, GianmariaPelletier, J.M.D’Amato, MichelePelli, StefanoDa Fonseca, Gláucio SoaresPellis, AlessandroDa Gomes, Maria IdáliaPellone, LorenzoDa Silva, Pedro RaposeiroPeña, Victor PachecoDa Silva, Alisson KwiatkowskiPeng, JianDabagov, Sultan B.Penuelas, JoséDabiri, MohammadPenz, Florian MarkusDabrowska, DominikaPepelanova, IliyanaD'accolti, LuciaPeppel, TimDachineni, RakeshPerale, GiuseppeD'Adamo, IdianoPerdahcioglu, SemihDadfarnia, MohsenPereira, Mauro FernandesDadkhah, MehranPereira, OctavioDahal, JibaPereira, AntónioDahal, BishnuPereira, Ana LuisaDahal, TulashiPereira, EulaliaDahle, SebastianPereira, DoloresDai, WeiPereira, Nelson A. M.Dai, JianmingPerera, SudanthaDai, YingPerera, NoelDaiki, HatanakaPérez, GloriaDailly, JulianPerez-Acebo, HeribertoDalconi, Maria ChiaraPerez-Delgado, Maria-LuisaDalessandri, DomenicoPerez-Gavilan, ArianeD'Alessandro, AntonellaPérez-Köhler, BárbaraDalkiranis, Gustavo GonçalvesPérez-Soriano, Eva M.Dallago, MichelePeriasamy, AnbuDalmoro, AnnalisaPeric, MatoDÁmato, RobertoPérigo, Élio AlbertoDamjanović, DarkoPerilli, StefanoDamma, DevaiahPerissutti, BeatriceDammak, LasaadPerkins, GregDammaschke, TillPermin, DmitryD'Amora, UgoPernyeszi, TímeaD'Amore, AlbertoPerreault, PatriceDan, DobrotaPerreault, FrancoisDana, StancekovaPerrella, MicheleDa'na, EnshirahPerrot, ArnaudDancette, SylvainPerrotta, AlbertoD'Andrea, AntonioPerrotti, VittoriaDaneshvar, HosseinPerry, NicolaDangol, ManitaPersson, Karl-MagnusDaniel-da-Silva, AnaPerteghella, SaraDAnte, SilviaPerumal, SugunaDanti, SerenaPerumal, PriyadharshiniDao, Van-DuongPerveen, AsmaDarvell, Brian WPesika, Noshir S.Das, HrishikeshPestov, AlexanderDas, PradipPetala, NatasaDas, SujitPetcu, CristianDash, BirajaPeter, Emanuel KarlDatcheva, MariaPeter Amalathas, AmalrajDatta, KaustuvPeters, William K.Daugevičius, MykolasPetersen, ElijahDaus, AlwinPeterson, Dominic S.Davari Esfahani, M. MahdiPetit, LaeticiaDavim, J. PauloPetkova, DianaDavis, GrahamPetráková, VladimíraDavis, PaulPetrella, AndreaDavoudi, MasoumePetrescu, CatalinDavoudinejad, AliPetrič, MitjaDavydova, Elena S.Petrosanu, Dana-MihaelaDawes, LesPetrov, PetеrDawn, ArnabPetrov, PavelDawson, James A.Petrović, MarijaDawson-Andoh, Benjamin E.Petrů, MichalDbouk, TalibPetrus, JosefDe Aguiar, José BarrosoPetukhov, Dmitriy I.De Almeida, Jose Carlos MartinsPeys, Arnede Araújo Nobre, MiguelPezzato, LucaDe Aza, Piedad N.Pezzuolo, AndreaDe Biase, AlbertoPfleger, JiriDe Bonis, AngelaPhan, Hoang-PhuongDe Boor, JohannesPhan, KhaDe Castro, AlexandrePhilipose, UshaDe Cos, ElenaPhillips, KatherineDe Domenico, DarioPhoenix, S. LeighDe Falco, GianluigiPhu Nguyen, VinhDe Finis, RosaPhuah, Xin LiDe Freitas, Elisabete FragaPhung, Luong-VietDe Haart, L.G.J.Phung, Quoc TriDe La Hoz, AntonioPiasecki, MichalDe Lacalle, Luis N. LópezPiatkevich, Kiryl D.De Lima Jr., Mauricio M.Picas, Josep A.De Luca, LuigiPiccinelli, FabioDe Luca, AlessandroPiccioni, Mario DanieleDe Luca, PierantonioPiccirillo, ClaraDe Luna-Bertos, ElviraPichór, WaldemarDe Melo-Diogo, DuartePicó, RubénDe Napoli, LuigiPieczyska, Elzbieta A.De Oliveira, Luiz Antonio PereiraPiedade, Ana PaulaDe Oliveira Junior, MarcosPielichowska, KingaDe Rosis, AlessandroPielichowski, KrzysztofDe Santis, RobertoPieniak, DanielDe Santis, MichelePierard, OlivierDeac, CristianPierini, FilippoDebiec-Andrzejewska, KlaudiaPierpaoli, MattiaDeboucha, WalidPierre, AlexandreDecroly, AndréPierściński, KamilDedova, TatjanaPietras, AdamDegiacomi, Matteo T.Pietrikova, AlenaDegli Esposti, MicaelaPietrzyk, PiotrDehghan, AmirPietrzyk, BozenaDehghanghadikolaei, AmirPijls, BartDeja, MariuszPilarczyk, WirginiaDejam, MortezaPilehvar, ShimaDel Gallo, MddalenaPilipovic, AnaDel Giudice, FrancescoPilo, RaphaelDel Hougne, PhilippPilot, RobertoDel Hoyo Martínez, CarmenPiluso, SusannaDel Toro, Eva M. GarcíaPilyugina, EkaterinaDel Val, JesúsPimenov, Danil YuDel Vecchio, CiroPina, SandraDel Zoppo, MartaPineda, EloiDelattre, CédricPineda, PalomaDelbe, KarlPiñero, ManuelDelebarre, ChristophePingarrón, JoséDelfanazari, KavehPingitore, NicholasDelgado Ruiz, RafaelPinho, LuísDell’Agli, GianfrancoPinho, DianaDell’Isola, MarcoPinkerton, AndrewDelle Piane, MassimoPinto, FulvioDelsaute, BricePinto, DeesyDelucchi, MarinaPinto, Gustavo FilipeDemertzis, KonstantinosPinto, Maria Isabel MoitaDemetrescu, IoanaPinto Da Silva, LuísDemey, HaryPiotrowski, PiotrDemir, Ali GökhanPiozzi, AntonellaDemirci, IbrahimPiracha, AfaqDeng, GuochuPirjan, AlexandruDeng, NengxiuPirri, AngelaDeng, XiaoranPísařík, PetrDenisiewicz, ArkadiuszPisarska, JoannaDeo Malviya, KirtimanPisarski, W.a.Depciuch, JoannaPiscitelli, FilomenaDepczynski, WojciechPistone, AlessandroDerakhshan, HosseinPiszczek, PiotrDeretzis, IoannisPitarch Roig, Angel MiguelDerkatch, SvetlanaPitcher, EricDermol-Černe, JanjaPiticescu, Radu RobertDeshpande, AnaghPitilakis, AlexandrosDesirena, HaggeoPitois, OlivierDettori, MarcoPitsikalis, MarinosDEVEL, MichelPizzolanti, GiuseppeDevelos-Bagarinao, KatherinePlachá, DanielaDevine, DeclanPlakas, KonstantinosDewa, Rando TunggaPlazl, IgorDey, VikramPlekhov, OlegDhakal, DipeshPloss, BerndDhaliwal, Gurpinder SinghPlutino, Maria RosariaDhaliwal, GurjotPo, GiacomoDhamdhere, AjitPobelov, IlyaDharavath, SrinivasPoccia, NicolaDhiman, SaurabhPochard, IsabelleDhindsa, Kulwinder SinghPoddel'sky, AndreyD'hooge, Dagmar R.Podgórski, JerzyDI, ChukovPodgursky, VitaliDi Bartolomeo, AntonioPodsiadły-Paszkowska, AgataDi Bona, GianpaoloPoehl, FabianDi Buduo, ChristianPoelman, DirkDi Caprio, FrancescoPoggio, ClaudioDi Cocco, VittorioPohl, MichaelDi Corato, RiccardoPokhrel, MadhabDi Gianfilippo, RiccardoPolaczyk, PawelDi Gioacchino, FabioPolanski, MarekDi Girolamo, RoccoPolanský, RadekDi Maio, LucianoPolat, Emre OzanDi Marco, AlessandroPoletti, Maria CeciliaDi Martino, PieraPolice, Anil Kumar ReddyDi Mascio, PaolaPolimeni, AntonellaDi Noia, Luigi PioPolimeno, AntoninoDi Salle, AnnaPolitakos, NikolaosDi Sarli, ValeriaPolitano, AntonioDi Sarno, LuigiPolitano, Grazia GiuseppinaDi Stasio, DarioPolito, LauraDi Turo, FrancescaPolivtseva, SvetlanaDiana, RositaPolozov, IgorDias, JulianaPomarede, PascalDias, George JayanthaPoncsák, SándorDiaz, EsperanzaPondelak, AndrejaDíaz, José AntonioPonikiewski, TomaszDíaz Fernández, BelénPons, OriolDíaz-Álvarez, AntonioPons, DirkDiaz-Maurin, FrançoisPonticelli, Gennaro SalvatoreDiaz-Real, JesusPoon, JosephDiaz-Rodriguez, PatriciaPop, OanaDiban, NazelyPopa, LacramioaraDickey, MichaelPopescu, VasilicaDiederichsen, KylePopescu, AndreiDiego, Ignacio GarcíaPopescu, SimonaDiego P., OyarzúnPorojan, LilianaDietrich Zagonel, FrancielePorras Amores, CésarDiko, PavelPortal, Natalie WilliamsDikova, Ts. D.Porzionato, AndreaDilip, DepanPosadas, PilarDilonardo, ElenaPospíšilová, ŠárkaDimakis, NicholasPostnikov, AndreiDimakopoulos, YannisPostnikov, PavelDimitratos, NikolaosPotapov, AndreiDimitrellou, DimitraPotgieter, HermanDimitri, RossanaPou, JuanDimitrievska, MirjanaPoulia, AnthoulaDimitrov, BorislavPoullain, PhilippeDimos, KonstantinosPouraliakbar, HesamDimter, SanjaPouranian, M. RezaDinca, Paul PavelPourrahimi, Amir MasoudDINESCU, GheorghePous, NarcisDing, Yung-ChingPoveda Bautista, ElisaDing, ZhaoyangPoveda-Martínez, PedroDing, H.F.Povoden-Karadeniz, ErwinDing, JianxunPovolotskiy, Alexey V.Ding, Wei DanPoyato, RosalíaDinh, ToanPozdniakov, AndreyDini, DaniloPozegic, ThomasDiogo, PatríciaPozzolini, MarinaDiomede, FrancescaPrabakaran, MayakrishnanDionysopoulos, DimitriosPrabhakaran, DharmalingamDipu, Arnoldus LambertusPrabhudev, SagarDirè, SandraPradhan, Sangram K.Dirk, HenrichPradhan, Dhiren K.Distaso, MonicaPrado-Gonjal, JesúsDixon, DavidPrados-Frutos, Juan-CarlosDiyaroglu, CaganPrados-Privado, MaríaDoagou-Rad, SaeedPrajer, MarekDobák, SamuelPrajzler, VaclavDobiszewska, MagdalenaPramanik, BrahmanandaDobrotă, DanPramanik, AlokeshDobrovodský, JozefPramudita, Jonas A.Dobrzyński, MaciejPranantyo, DickyDochshanov, AldenPrasadh, SomasundaramDodero, VeronicaPrashanth, Konda GokuldossDogossy, GáborPrashar, SanjivDogra, PrashantPrates, PedroDohnalová, ŽanetaPraticò, Filippo GianmariaDoi, KotaroPrazuch, JanuszDolata, AnnaPredoi, DanielaDoležal, PavelPrehl, JanettDolocan, AndreiPrelipceanu, MariusDomagała, LucynaPresto, SabrinaDománková, MáriaPresz, WojciechDomènech-Gil, GuillemPrevitali, BarbaraDomenici, ValentinaPrieto, Rafel M.Domine, Marcelo E.Prieto, Andrés J.Domski, JacekPrimus, Carolyn M.Doñate, Cristina MartínPrincipe, MariaDonchev, AlexanderPrisco, UmbertoDong, ChengyuanPristovsek, MarkusDong, XufengPriya, PikeeDong, QiaoProcek, MarcinDong, XianghuaiProchazka, MarekDong, HongbiaoProchazka, VitDong, JianghuiProsek, TomasDonic, TiborProslier, ThomasDonik, CrtomirProsviryakov, Alexey S.Donnermeyer, DavidProto, AndreaDonnini, JacopoPrudenzano, FrancescoDoostmohammadi, HamidPruna, AlinaDorafshan, SattarPruncu, CatalinDore, ElisabettaPrusa, FilipDoria, AndreaPrusov, EvgenyDorin, ThomasPryamikov, AndreyDorner-Reisel, AnnettPrywer, JolantaDorozhkin, Sergey V.Przestacki, DamianDorp, Dennis VanPrzybyłek, MaciejDos Santos, Leonardo H. R.Pshyk, AleksandrDos Santos-García, Antonio JuanPszczola, MarekDow, Hwan SooPucci, Monica FrancescaDragan, ValeriuPuchalski, MichałDraganovska, DagmarPuga, HélderDraghi, LorenzaPuglia, DeboraDragoman, MirceaPuglisi, Francesco MariaDragutan, ValerianPui, AurelDrawin, StefanPuiggalí, JordiDreier, ThomasPujadas, PabloDrelich, JaroslawPuli, Venkata SreenivasDrewniak, SabinaPulido, DavidDrexler, AndreasPullano, SalvatoreDrienovsky, MarianPulyalina, AlexandraDrissi-Habti, MonssefPung, Aaron J.Drobná, HelenaPunzo, FrancescoDroulias, SotirisPuppulin, LeonardoDrozdenko, DariaPurcar, VioletaDrstvenšek, IgorPurgel, MihályDruesne, FredericPushilina, NataliaDrzazga, MichałPustan, MariusDrzeżdżon, JoannaPuszka, AndrzejDu, HongjianPuszkiel, JuliánDu, XushengPutkonen, MattiDu, Jia-ChongPutland, Rosalyn L.Duan, BinPutlayev, ValeryDuarte, IsabelPyatina, TatianaDuarte Campos, Daniela F.Pyatnov, MaximDuarte Marafona, JoséPyl, LincyDub, Alexei V.Pyo, SukhoonDubé, MartineQasim, SaadDubenko, IgorQayyum, FaisalDubey, RichaQi, ZenanDubiel, BeataQi, ZhiyuanDucharne, BenjaminQi, ZhuyuanDuchesne, MarcQian, GujieDuchet-Rumeau, JannickQian, Jin-yuanDuchi, SerenaQiang, ZheDuchosal, ArnaudQin, YihengDucobu, FrançoisQiu, QiwenDudás, ZoltánQiu, MingboDudescu, Mircea CristianQiu, JishenDudin, PavelQu, MuchaoDudina, Dina V.Qu, ShengguanDudziak, TomaszQuan, GaofengDuisit, JeromeQuarto, MariangelaDul, SithiprumneaQuattrocchi, AntoninoDulal, RajendraQueffélec, ClémenceDulska, AgnieszkaQuerales-Flores, JoseDulski, MateuszQuik, Joris T.K.Duma, Virgil-FlorinQuintana, AlbertoDumas, LudovicQureshi, TanvirDumbrava, AncaRabadán-Ros, RubénDumitriu, CristinaRabbani, VahidDummer, Nicholas F.Räbiger, DirkDumur, FrédéricRabu, PierreDuncan, OllyRace, MarcoDunlop, ElaineRackauskas, SimasDunne, NicholasBabuta, RoxanaDunne, PeterRac-Rumijowska, OlgaDuque, Johan S.Racz, GabrielDuQuesnay, DavidRaczkiewicz, WiolettaDurães, LuísaRadek, NorbertDurand, BastienRadonic, VasaDurán-Peña, María JesúsRadosław, JasińskiDurejko, TomaszRadtke, MariuszDurndell, LeeRadtke, AleksandraD'Urso, GianlucaRadu, DorinD'Urso, SebastianoRǎducanu, DoinaDuta, LiviuRadulescu, CristianaDutkiewicz, Maciej RobertRadwański, KrzysztofDutta, BiswanathRadziszewski, PiotrDutta, GaurabRaeis-Hosseini, NiloufarDutta, AniruddhaRafiee, MohammadDutta, Ravi ChandraRagab, MohyeldinDutta, AbhishekRagab, KhaledDuvallet, Tristana Y.Raghavan, NarendranDvorský, TomášRahbar-Rastegar, ReyhanehDyatkin, BorisRahier, HubertDyl, TomaszRahimi, MohammadDymond, MarcusRahman, Md. MahbuburDymshits, OlgaRahman, MostaqurDyskin, ArcadyRahman, MuhibDzantiev, BorisRahman, MuhibUrDziedzic, ArkadiuszRahman, Mohammad ShafiqurDziekońska-Kubczak, UrszulaRahman Rashid, Rizwan AbdulDziewit, LukaszRahmani, RaminDzioba, IhorRahme, KamilDzionk, StefanRaicopol, Matei D.Dziubińska, AnnaRaimondo, MariarosaDziurdzia, PiotrRaimondo, MarialuigiaDziurka, DorotaRaith, StefanDzolic, ZoranRaja, JayendiranEbendorff, HeikeRajaraman, SwaminathanEberle, PatricRajca, MariolaEberlein, RobertRajnak, MichalEbrahimi, AminRaju, Muppala N. P.Ebrahimi, NafisehRaju, KatiEbralidze, Iraklii I.Rakhshbahar, MohammadEchterhof, ThomasRallini, MarcoEchtermeyer, AndreasRalston, EmilyEcker, MelanieRamachandran, RoshiniEdström, DanielRamakrishna, Shivaprakash N.Eftekhari, AliRamakrishnan, SayanthanEfthymiadis, PanosRamakrishnan, SathishEgan, Paul F.Ramalingame, RajarajanEgberts, PhilipRamamoorthy, Sunil Kumar LindströmEgner, HalinaRamanavicius, ArunasEgorin, AndreiRamandi, Hamed LameiEgorkin, VladimirRamani, AlwarEguchi, KeiRamasamy, MohankandhasamyEhrmann, AndreaRamasamy, ElamparuthiEhsan, MostafaviRamezani, MajidEhtemam Haghighi, ShimaRamos, JavierEickenscheidt, MaxRamos Gavilán, Ana BelénEinkauf, Jeffrey D.Ramseyer, Chris C.Eiras Fernández, JesúsRana, Abu Ul Hassan SarwarEisenträger, JohannaRanachowski, ZbigniewEisinas, AnatolijusRanachowski, PrzemysławEkielski, AdamRanc, VáclavEklund, PerRanjbar, NavidEklund, Sven E.Ranjkesh, AmidEl Sayed, SamehRankin, Rees B.Elahi, HassanRao, AshutoshElambasseril, JoeRao, HarishElamin, KhalidRaposio, EdoardoElbert, JohannesRaposo, MariaElbestawi, MohamedRappel, HusseinEl-Bialy, TarekRárová, LucieEldridge, JeffreyRashid, Rizwan RahmanElena, Tiuc AncuţaRashid, Mohammad MamunurElert, KerstinRashid, KhalidElgali, IbrahimRashidi, Mohammad MehdiEl-hadi, Ahmed M.Raspet, RichardElhajjar, RaniRasskazov, IliaEl-Hakim, MohabRassõlkin, AntonElhattab, AhmedRastogi, Vibhore KumarEliades, GeorgeRatajczak, MonikaElías-Zúñiga, AlexRath, PunyaslokEliche Quesada, DoloresRathinam, KarthikEligiusz, PostekRathousky, JiriElkaseer, AhmedRatova, MarinaEl-Kazzi, SalimRaty, Jean-Yves RatyEl-kharbachi, AbdelRatzke, MarkusElkoun, SaidRaucci, UmbertoEllendt, NilsRaucci, Maria GraziaEllinas, KosmasRaudino, AntonioElmahdy, AhmedRault, FrançoisElmouwahidi, AbdelhakimRaut, PrasadElRashedy, RemahRautkari, LauriElsayed, AdhamRauwel, ErwanElzohairy, AymanRauwel, ProtimaEmad, Muhammad ZakaRavaioli, StefanoEmadi, ArezooRavasio, ChiaraEmami, AnahitaRavelli, DavideEnǎchescu, MariusRavindra, NuggehalliEndruweit, AndreasRavindra, Nuggehalli M.Enesca, Ioan AlexandruRavindran, SurajEnfedaque, AlejandroRazavi, JavadEngel, DieterRazi, HajarEngler, OlafRebaioli, LaraEngwall, AlisonRebelo, RitaEnikeev, NarimanReber, ChristianEnkhsaikhan, BoldsaikhanRecek, NinaEnomoto, MasatoReddy, M. Siva PratapEnrico, Gherlone FeliceReddy, M. VenkatashamyEnríquez Pérez, EstherRedwing, Joan M.Enyashin, AndreyRegel-Rosocka, MagdalenaEpasto, GabriellaRegev, MichaelEpshtein, SvetlanaReggiani, BarbaraErceg, AleksandarRego, GasparErck, RobertRegoutz, AnnaErdakov, Ivan NikolaevichReguero, MarErdélyi, JánRegulska, ElzbietaErdem, EmreRehel, KarineEremin, ArtemRehm, Thomas H.Eric Kast, RichardRehman, Zeeshan UrEriksen, René LyngeReilly, AidanErlebach, AndreasReinke, Gerald H.Ermoline, AlexandreReinosa, Julián JiménezErrandonea, DanielReis, Paulo Nobre Balbis DosErtman, SławomirReiter, LexErythropel, HannoReiterman, PavelEsashi, MasayoshiRekasi, MarkEscobedo-Lucea, CarmenRen, ZhaoyuEscoda, LluïsaRen, YanfangEscola, José MaríaRen, LingEsfahani Monfared, YasharRen, JamesEsin, Vladimir A.Ren, KaixuanEsmaeely Neisiany, RasoulRen, GuofengEsmeryan, KarekinRenaudin, GuillaumeEspinach, Francesc XavierRene, EldonEspinosa, ChristineRenner, FrankEsposito, FlavioRepa, Kristen StojakEsposito, LucaRepetto, Maria PiaEsposito, SerenaRequicha, JoãoEstelrich, JoanRescalvo, FranciscoEstêvão, João M.C.Resnina, NataliaEstokova, AdrianaRestrepo Baena, Oscar JaimeEttayapuram Ramaprasad, Azhagiya S.Restuccia, PaoloÉva Eszter, LublóyRevenant, ChristineEvans, Jack D.Reverberi, Andrea P.Everaerts, JorisRevuru, Rukmini SrikantEvtugyn, GennadyRew, YounhoEzzeddine, ZeinabReyes, AaseF. Vidor, FábioReyes-Ortega, FelisaFabbri, PaolaRezvani, EhsanFabianowski, WojciechRhee, InkyuFabregue, DamienRho, JunsukFacchini, FrancescoRiad, KhettabiFacconi, LucaRibeiro, JoãoFadeeva, IrinaRibeiro, Carlos A. SilvaFaggio, GiulianaRicca, MichelaFaisal Haider, MohammadRicciardi, Maria RosariaFajín, José L. C.Ricciardi, GiuseppeFakhrullin, Rawil F.Ricciotti, LauraFalchetto, Augusto CannoneRice, AnthonyFales, AndrewRichard, BenjaminFaleschini, FloraRichards, Dylan JackFaley, MichaelRichert, MariaFallah, Arash S.Richeton, ThiebaudFaller, Lisa-MarieRichter, CarstenFan, JizhouRichtera, LukášFan, WeiRider, Andrew N.Fan, ZhongmingRiedl, HelmutFan, ZhengjieRiesco Munoz, GuillermoFan, QiaohuiRiess, GisbertFan, DapingRighini, GiancarloFan, WeizhengRiha, Shannon C.Fancher, ChrisRijckaert, HannesFanelli, PierluigiRimola Gibert, AlbertFang, RunchenRimondini, LiaFang, GangRinaldi, MariannaFang, XudongRingdalen, Inga GudemFang, MingxiRinkevich, AnatolyFangrat, JadwigaRíos-Santos, Jose VicenteFantoni, FrancescaRipamonti, DarioFantuzzi, NicholasRischka, KlausFaraguna, FabioRistić, MiraFaraldos, MarisolRitter, HelmutFare, SilviaRiu, Doh-HyungFareed, AmberRivadeneyra, AlmudenaFargnoli, MarioRiveiro, AntonioFarokhi, HamedRivera, Francisco F.Farquhar, AnnaRivera-Gómez, CarlosFarràs, PauRivero, Pedro J.Farrokh Ghatte, HamidRiveros, Guillermo A.Farronato, GiampietroRiviera, Pier PaoloFarshbaf, SepidehRizal, ConradFaruqi, M. A.Rizzi, Gian AndreaFarzampour, AlirezaRizzi, EgidioFarzanian, KhashayarRizzo, RoccoFateri, SinaRizzo, FabioFatouros, Dimitrios G.Rizzolio, FlavioFaulstich, MartinRo, InsooFauzi, IlianaRoa Rovira, Joan JosepFavi, ClaudioRoberto, DominiqueFavre, JulienRobinson, Jacob T.Fayaz, MohammadrezaRobinson, PeterFayazfar, HaniyehRobisson, AgatheFazakas, EvaRobusto, FrancescoFazeli, FatehRoccaforte, FabrizioFedorko, GabrielRocchitta, GaiaFedorov, Vladimir E.Rocha, FernandoFedorov, PavelRocha, Iuri B. C. M.Feito, NorbertoRockett, AngusFeld, ArturRodeghero, ElisaFeldman, DaleRodič, PeterFeldmann, Maik WilhelmRodrigue, DenisFeldshtein, Eugene ÉRodrigues, Célia F.Felgueiras, Helena P.Rodriguez, AngelFelho, CsabaRodriguez, NoelFeliú Jr., SebastianRodriguez, PilarFelner, IsraelRodriguez, Jose P.Felsmann, ClemensRodriguez, Aurelio HierroFeng, HongboRodríguez, J. Antonio TraviesoFeng, ChuangRodriguez Diaz, Camilo ArturoFeng, Sheng-WeiRodríguez Saiz, ÁngelFeng, PeizhongRodríguez-Alabanda, ÓscarFeng, YixuanRodríguez-Alloza, Ana MaríaFeng, YongjunRodriguez-Correa, CatalinaFeofilov, ArtemRodríguez-Lorenzo, LuisFerdous, WahidRodríguez-Lozano, Francisco JavierFerenc, KristályRodríguez-Tinoco, CristianFernandes, Maria HelenaRofouie, PardisFernandes, FábioRogalski, GrzegorzFernandes, Susete NogueiraRogers, Simon A.Fernandes, FranciscoRoggendorf, HansFernández, Juan PedroRoggendorf, Matthias JFernández, María JesúsRogl, GerdaFernández, José MariaRogolino, PatriziaFernández-Canteli, AlfonsoRogovoy, Anatoly A.Fernandez-Garcia, RaulRoh, Jong-wookFernández-García, MartaRoh, ChanghyunFernandez-Lafuente, RobertoRoh, Jea-SeungFernandez-Roldan, Jose AngelRojas, Jose I.Fernandez-Vidal, Severo RaulRojas-Hernandez, Rocío EstefaníaFerrandez-Villena, ManuelRojek, JerzyFerrari, PieroRojo, RosaFerrari, MicheleRojo, Maria AngelesFerraro, StefanoRokosz, KrzysztofFerraz, Emanuela PradoRoldán, MarceloFerré-Borrull, JosepRolinski, EdwardFerreira, Manuel MarquesRomagnoli, ManuelaFerreira, BárbaraRoman, KoernerFerreira, FilipeRomán-Martínez, M.C.Ferreira, Jose M.F.Romanowicz, PawełFerrer, BelenRomanowicz, PawelFerrero, Guillermo Á.Románszki, LorándFerretti, ElenaRomar, HenrikFerrotti, GildaRomero, PedroFerry, LaurentRomero, IsabelFeteira, AntonioRomero, Pablo E.Feugeas, FrancoiseRonca, AlfredoFey, TobiasRončević, Ivana SˇkugorFiala, LukášRondinini, SandraFicek, MateuszRong, LiFicker, TomasRoppolo, IgnazioFidalgo, MariaRosa, InêsFidan, IsmailRosa, ViniciusFidel, Salas VicenteRosado, CatarinaFiedler, ThomasRoscow, JamesFigueira, RitaRosencrantz, Ruben R.Figueira, Ana CristinaRosenzweig, DerekFigueira, Rita BacelarRosicka-Kaczmarek, JustynaFigueiras, FabioRoskosz, MaciejFigueiredo, José L.Rösner, BenediktFigueiredo, Stefan ChavesRossi, ManuelaFilarowski, AleksanderRossi, FedericoFilice, SimonaRossi, René M.Filippov, AndreyRossi, ClaudioFilová, EvaRossignol, JulienFine, James BurkeRost, Christina M.Finney, Eric E.Roszak, JoannaFinšgar, MatjažRoters, FranzFintová, StanislavaRoth, Stephan V.Fiorenza, RobertoRoth, JohannesFiorenza, PatrickRoulia, MariaFiori, StefanoRoupcová, PavlaFiorillo, LucaRousakis, TheodorosFirlak, MelikeRousseau, MartheFischer, Franz DieterRovatti, LudovicaFischer, Hartmut R.Rovnaník, PavelFlege, StefanRowthu, SriharithaFleury, BlaiseRoy, KasturiFlorczak, BogdanRoy, DhruvajyotiFlorczyk, StephenRoy, MatthewFlorea, MihaelaRoy, Marie E.Florence, LequienRoy, SougataFlorentin, EricRóżalska, BarbaraFlores, Marta RoigRozman, NejcFlores Ituarte, IñigoRozumek, DariuszFlores-Alés, VicenteRozynek, ZbigniewFlores-Colen, InesRubano, AndreaFlorescu, MonicaRuben, PereiraFlorian Baron, CamiloRubio, Leire RuizFlury, SimonRubio-Cintas, Maria DoloresFlynn, J.M.Rubio-Marcos, FernandoFocaroli, StefanoRucińska, TeresaFodor, CsabaRucka, MagdalenaFologea, DanielRudakova, AidaFonseca, AnaRudawska, AnnaFontana, Marc D.Rudolf, RebekaFontanari, VigilioRuello, Maria LetiziaFontanesi, ClaudioRuffin, PaulFontinha, Isabel R.Ruffino, FrancescoFoot, PeterRui, XianhongForaboschi, PaoloRuiz, AmaliaFormela, KrzysztofRuiz, Harold StevenFornaguera, CristinaRuiz-Cabello, Francisco Javier MontesFornaini, CarloRukavina, TatjanaFornalczyk, AgnieszkaRukavina, Marija JelcicFornasiero, PaoloRukavishnikov, VictorFornell, JordinaRumyantseva, MarinaForner, LeopoldoRunacres, Mark C.Forsey, Alexander N.Runčevski, TomčeForster, Aaron M.Rung, StefanFörsth, MichaelRunge, Jude MaryFortea-Verdejo, MartaRupp, FrankFortenberry, RyanRüscher, ClausFortino, StefaniaRussell, Alan M.Fortmann, CharlesRusso, VincenzoFortunati, IlariaRusso, SaverioFoti, DoraRusso, PietroFottovati, BehzadRusso Spena, PasqualeFox, PeterRusu, Laura CristinaFraboni, BeatriceRutkowski, PawełFracchia, ElisaRuys, AndrewFraddosio, AguinaldoRůžek, RomanFragalà, M. E.Ryan, James G.Fragassa, CristianoRybak, JarosławFrança, RodrigoRybakov, Kirill I.Franceschini, GiordanoRybakov, K.I.Francesconi, LucaRybarczyk, PiotrFrancés-Soriano, LauraRybiński, PrzemysławFrancia, CarlottaRydosz, ArturFranco, Francesco DiRydz, JoannaFranco, LourdesRydzkowski, TomaszFrançois, Buyle-BodinRyl, JacekFrancolini, IolandaRyou, HeonjuneFrangini, StefanoRyou, Jae-SukFrank, IrmgardRyparova, PavlaFrankcombe, TerryRys, DawidFranus, WojciechRytlewski, PiotrFraternali, FernandoRytöluoto, IlkkaFratini, FabioRyu, JehwangFrattini, DomenicoRyu, Seung YoonFreddi, FrancescoRyu, SeongwooFresco-Cala, Beatriz MaríaS. Esmaeeli, HadiFreudenberger, JensSaadatnia, ZiaFriák, MartinSaalwachter, KayFricke, WolfgangSabatini, ValentinaFrieß, MartinSabban, AlbertFrigura-Iliasa, Flaviu MihaiSabhachandani, PoojaFröhlich, EleonoreSablé, SophieFronteraIPUS, JHON J., AntonioSaboori, AbdollahFroriep, Ulrich P.Sacco, OlgaFruggiero, FabioSacco, RiccardoFryda, LydiaSaccomandi, PaolaFryska, SebastianSachelarie, LilianaFu, FengSacher, EdwardFu, TengfeiSachs, PatrickFu, ZhezhenSadegh, HamidrezaFu, QiangSadeghifar, HamidrezaFujieda, TadashiSadek, MohammadFujii, SatoshiSadowski, ŁukaszFUJII, GarudaSadrzadeh, MohtadaFujii, Mami N.Saeedifar, MiladFujioka-Kobayashi, MasakoSaeedipour, MahdiFujita, TakeshiSáez, Elena RodríguezFujita, Shin-ichiroSáez Blázquez, CristinaFujiwara, AkihikoSafa, MeerFukuda, TakeshiSafaei, Mohammad RezaFulvio, Pasquale FernandoSafarian, JafarFunari, Marco FrancescoSaffari Pour, MohsenFuoco, TizianaSafonov, AlexanderFurimsky, EdwardSaghaian, Sayed M.Furlani, ErikaSah, Santosh PrasadFurness, JamesSaha, Amit KumarFuruta, MamoruSaha, Ashish KumerFuso, FrancescoSaha, BiswadeepFydrych, DariuszSaha, SwarnaGabdullin, NikitaSaharan, LalitaGabriela, LewinskaSahin, Furkan E.Gabriel-Alexandru, ConstantinSahoo, SanjubalaGabryś, KatarzynaSahu, Saura C.Gadaguntla Radhakrishnan, BalachandranSaid, NeveenGadea, Jose MariaSaid Schicchi, DiegoGadikota, GreeshmaSaidaminov, MakhsudGadisa, Abay DinkuSaidani, MessaoudGaffet, EricSaielli, GiacomoGagani, AbedinSaikko, VesaGagarin, AlexanderSaiko, GuennadiGagliano, AntonioSaimoto, AkihideGagliardi, FrancescoSain, SunandaGahleitner, MarkusSaini, NaurangGain, Asit KumarSaini, ShikhaGaitzsch, JensSainz-Díaz, IgnacioGajdošová, KatarínaSaito, MakinaGajewski, MarcinSaive, RebeccaGajjar, ChiragSajkiewicz, PawełGala, Rikhav PSakagami, KimihiroGalamboš, MichalSakai, TakenobuGalan, JesusSakai, JoeGalan, IsabelSakar, MohanGalanakis, IosifSakharova, NataliyaGalanis, NikolaosSakkas, KonstantinosGalao, OscarSakon, TakuoGalassi, CarmenSakthivel, TamilselvanGaletz, MathiasSakuraba, MasaoGalgalikar, RohanSala, RobertoGalietti, UmbertoSalach, JacekGalizia, PietroSałaciński, TedeuszGalkiewicz, JaroslawSalado, ManuelGalkin, MaximSalameh, ChrystelleGalla, StanislawSalami, Mohammad RezaGallardo-López, AngelaSalapare, Hernando IIIGallego, AntolinoSalas, CarlosGallina, AlbertoSalasi, MobinGalopin, NicolasSałasińska, KamilaGalvão, IvanSalaün, FabienGambardella, AlessandroSalbach-Hirsch, JulianeGambarini, GianlucaSaleh, Mohamed NasrGamnitzer, PeterSalehi, SaeedGamsjäger, ErnstSalehinia, ImanGan, YongSalerno, MarcoGanczarski, Artur WładysławSalguero, Tina T.Gangireddy, SindhuraSalguero, JorgeGangwar, KapilSaliani, Saeed S.Ganta, DeepakSaliev, TimurGao, ZheSaligram, ShreyasGao, XuenongSalikhyanov, DenisGao, ShuangSallam, Khaled A.Gao, XiaojianSalmas, Constantinos E.Gao, TongSalmi, MikaGarapati, NagasreeSalmina, AllaGarašić, IvicaSalminen, AnttiGaray, HélèneSaltas, VassilisGaray, Javier E.Saluk-Bijak, JoannaGarbacz, AndrzejSalvati, EnricoGarbiec, DariuszSalvia, MichelleGarbovskiy, YuriySalzano De Luna, MartinaGarbrecht, MagnusSamadi-Dooki, ArefGarbuzenko, OlgaSamaitis, VykintasGarcez, Estela OliariSamal, SnehaGarcia, CristobalSamal, SangramGarcia, RosarioSamanta, Pabitra NarayanGarcía, DanielSamaras, IoannisGarcía, GregorioSamborski, SylwesterGarcía, LucíaSameer, HusamGarcía Calvo, José LuisSamek, LudovicGarcía Costa, Alicia LoretoSamia, BenmansourGarcía Diez, AnaSammelselg, VäinoGarcía Rodríguez, JuanSammler, FionaGarcía Ruiz, Francisco JavierSamoila, CornelGarcía-Astrain, ClaraSamokhvalov, AlexanderGarcia-Benett, AlfonsoSamouh, HamzaGarcia-Escorial, AsuncionSamuha, ShmuelGarcia-Gallego, SandraSanches, SandraGarcía-Granada, Andrés-AmadorSánchez, AntonioGarcía-Lastra, Juan MaríaSánchez, JavierGarcía-López, Elisa I.Sánchez Egea, AntonioGarcía-Martínez, Jesús-MaríaSánchez-Coronilla, AntonioGarcia-Pardo, JavierSánchez-García, DavidGarcia-Pomar, Juan LuisSanchis, Maria J.Garcia-Salaberri, Pablo AngelSander, ManuelaGarcia-Segura, SergiSandgren, EricGarcia-Taengua, EmilioSandu, ViorelGarcia-Vera, Victoria EugeniaSandu, Andrei VictorGargiulo, ValentinaSangiorgi, CesareGarma, TonkoSangiovanni, DavideGaroli, DenisSanin, Vladimir N.Gartia, Manas RanjanSanjuán, Miguel ÁngelGärtner, StefanieSanjurjo-Sanchez, JorgeGarvey, ChristopherSanjust, EnricoGas, PiotrSankaran, Kamatchi JothiramalingamGashti, Mazeyar ParvinzadehSan-Miguel, VeronicaGąska, AdamSannino, FilomenaGasparro, RobertaSansonetti, AntonioGatto, AndreaSantamaria, MonicaGauden, Piotr A.Santana, JoseGautam, BishnuSantangelo, Paolo E.Gavira, JoséSantangelo, SaveriaGavras, SarkisSantecchia, EleonoraGavrus, AdinelSantoro, SergioGawlik, JózefSantos, Luís PicadoGawlik-Dziki, UrszulaSantos, Joao MiguelGawronek, PelagiaSantos, AbelGawronska, ElzbietaSantos, BerthaGazizulina, AlbinaSantos, PauloGebicki, JacekSantulli, CarloGéczy, AttilaSantus, CiroGee, MarkSanyal, OishiGehlen, ChristophSanz Pont, DanielGeijselaers, Hubert J.M.Saoud, KhaledGelbstein, YanivSaparov, BayrammuradGelfi, MarcelloSapienza, VincenzoGeman, OanaSapudom, JiranuwatGenau, AmberSarabia, Aida Maria DiezGendelman, Oleg V.Sarafidis, CharalamposGenedy, MoneebSarafraz, Mohammad MohsenGeng, LinxiaoSaravanan, PrabakaranGeng, JinSarfraz, ShoaibGenova, TullioSarkar, DhrubaGentile, MarialuisaSarkar, Amit K.Gentile, PiergiorgioSarker, SubrataGeorge, Thomas F.Sarker, DyutiGeorgiev, Danko D.Sarli, Valeria DiGeorgieva, BilianaSarlin, EssiGeorgiou, EmmanuelSarma, SauravGeorgopanos, ProkopiosSarmad, ShokatGereke, ThomasSarti, ElenaGergely, JanosSartorio, CamilloGerislioglu, BurakSarvestani, Hamidreza YazdaniGerwin, WernerSarwar Rana, Abu ul HassanGesuele, FeliceSasajima, YasushiGhahari, Seyed AliSasaki, Jun-IchiGhanbari, AbbasSaseendran, VishnuGhane, MahdiSassani, AlirezaGhanmi, HelmiSastoque Pinilla, LeonardoGharam, AhmedSata, NorikoGharehveran, Mahsa ModiriSaternus, MariolaGhatak, KamalikaSato, TsutomuGhazijahani, Tohid GhanbariSato, KoichiGheni, Ahmed A.Sato, Shunsuke A.Gherardi, FrancescaSato, MitsunobuGheribi, Aimen GheribiSato, EiichiGhica, DanielaSato, TakuichiGhidelli, MatteoSaturnino, CarmelaGhirian, AlirezaSatyavolu, JagannadhGhodake, GajananSaulacic, NikolaGholizadeh-Vayghan, AsgharSaumer, MonikaGhosh, MonojSaunier, JohannaGhosh, SohamSavaniu, CristianGhosh, SubrataSavi, PatriziaGhoshal, SushantaŠavija, BrankoGhouse, ShaazSavu, DianaGhyngazov, Sergey AnatolievichSawada, HideakiGiacobbe, CarlottaSawpan, MoyeenuddinGiannakopoulou, Panagiota P.Sawtell, DavidGiannazzo, FilippoSaxer, FranziskaGiannella, VenanzioSayydi, NimaGiannetti, AmbraScajev, PatrikGiasin, KhaledScala, AngelaGibbons, Gregory JohnScamoni, FabioGibson, ChristopherScandurra, AntoninoGierada, MaciejScaramuzza, MatteoGierszewska, MagdalenaScarano, AntonioGierszewski, MateuszScarfato, PaolaGigante, Giovanni E.Scendo, MieczyslawGigli, MatteoŠčetar, MarioGil, JavierSchabowicz, KrzysztofGil, M.L.A.Schaedler, TobiasGilardi, ElisaSchäfer, HelmutGilardi, RaffaeleSchalk, NinaGillich, Gilbert-RainerSchartel, BernhardGillot, FrédéricScheffler, MichaelGilmore, JordonScheicher, Ralph H.Giorcelli, MauroScheinherrová, LenkaGiordani, MatteoSchenato, LucaGiorgetti, MarcoSchiavi, Pier GiorgioGiorgi, RodoricoSchiavone, GiuseppeGiorgia, GhiaraSchicchi, Diego SaidGiorgini, LorisSchierbaum, Klaus DieterGiorgio, IvanSchift, HelmutGiorleo, LucaSchiraldi, DavidGiovanardi, RobertoSchirhagl, RomanaGiovannetti, RitaSchirp, ClaudiaGiraud, StephaneSchlesinger, MarkGiraud, Marie NoëlleSchlick, GeorgGirginova, Penka I.Schlosser, ViktorGirish, RughooburSchmedake, Thomas A.Giró Paloma, JessicaSchmeer, SebastianGirolami, MarcoSchmid, JochenGirot, FranckSchmidova, EvaGiubileo, FilippoSchmidt, RainerGiudice, Giuseppe LoSchmidt, FranziskaGiudice, FabioSchmidt, JochenGiulia, FioravantiSchmidtchen, MatthiasGiunta, GaetanoSchmitt, RobertGiza, KrystynaSchmitz, ChristianGizdavic-Nikolaidis, MarijaSchnabel, ThomasGjokas, MargaritisSchneider, FelixGkoupidenis, PaschalisSchneider, EliaGładysz-Płaska, AgnieszkaSchoeppner, Rachel L.Glaser, BjörnSchönauer-Kamin, DanielaGlazar, IrenaSchowalter, MarcoGliniak, MaciejSchroeder, GrzegorzGliszczynski, AdrianSchroefl, ChristofGloria, AntonioSchubert, DavidGłowacki, MichałSchultheiss, JanGlushko, OleksandrSchulz, FlorianGlushkov, DmitriiSchulz, ChristianeGnade, BruceSchulze, MargitGnanasekaran, KarthikeyanSchulze-Tanzil, Gundula GesineGnesin, BorisSchwaminger, SebastianGniadek, MariannaSchwan, MarinaGniewosz, MałgorzataSchwartzentruber, JeffGnyba, MarcinSchwarze, MichaelGobber, Federico SimoneSchweiss, RuedigerGobeil Jr, FernandSchweizer, ThomasGöbel, LuiseSchwotzer, MatthiasGóbi, SándorSciumè, GiuseppeGockenbach, ErnstSciuto, Gaetano AntonioGoel, HimanshuScognamiglio, Pasqualina LianaGoel, SarikaScotti, AmericoGoenezen, SevanScotti, GianmarioGoerlitz, DetlefScribante, AndreaGogneau, NoelleScuro, CarmeloGoh, Kheng-LimScutelnicu, ElenaGohari, SoheilSeara-Paz, SindyGökce, BilalSebastian, VictorGola, ArkadiuszSebastian Marian, ZahariaGolabczak, Marcin AndrzejSecu, Elisabeta-CorinaGołaszewski, JacekSedai, Bhishma R.Golba, SylwiaSedelnikova, Mariya B.Golding, JonSedliačik, JánGoldsmid, H. JulianSeeholzer, LukasGołek, ŁukaszSeelam, Prem KumarGolewski, Grzegorz LudwikSefat, FarshidGolgovici, FlorentinaSegal, Vladimir M.Golkowski, MarkSegarra, MercéGolub, Mikhail V.Segawa, HiroyoGolub, IgorSegreto, TizianaGomez, Grace FelixSeguí, ConcepcióGómez de Salazar Caso de los Cobos, J.M.Seidenstuecker, MichaelGomez Melgar, SergioSeiersten, MarionGómez-Cámer, Juan LuisSeifan, MostafaGomez-Gil, VeronicaSeifert, GotthardGomez-Heras, MiguelSeifi-Mofarah, SajjadGómez-Polo, Miguel A.Seiler, PhilippGomez-Ruiz, SantiagoSeiner, HanušGomez-Sainero, Luisa MariaSek, SlawomirGómez-Soberón, Jose ManuelSekhar, JainageshGonçalves, Lídia M. D.Sekiguchi, YuGonçalves, RenatoSekiguchi, AtsukoGondro, JoannaSekikawa, JunyaGong, JiaweiSellars, JonathanGong, HaijunSemaltianos, Nikolaos G.Gong, PanSemboshi, SatoshiGonon, MauriceSemenova, Irina P.Gontarz, AndrzejSemisalova, AnnaGonzalez, ZoiloSemjonov, SergeyGonzález, SergioSemprini, GiovanniGonzález, María De Las NievesSen, SujatGonzález, IsraelSen Gupta, ArnabGonzález, Amador M.Sen Karaman, DidemGonzález, JoseSenatov, Fedor S.Gonzalez Vila, AlvaroSenderowski, CezaryGonzález-Cortés, AraceliSenetakis, KonstantinosGonzález-Fonteboa, BelénSeo, YongbeomGonzalez-Garcia, LolaSeok, HyunGonzalez-Gutierrez, JoaminSeok, OgyunGonzález-Martín, OscarSeol, Jae HunGonzález-Sánchez, M. I.Sepasgozar, Samad M.E.Gonzalo Orden, HernánSepe, RaffaeleGopalakrishnan, KothandamSepulveda, AbdonGopalan, SaianandSequeira, César A. C.Góra, JacekSerban, NicolaeGoral, MarekȘerban, Bogdan CătălinGordon, Wesley O.Serdechnova, MariaGoreham, ReneeSerenko, OlgaGorgieva, SelestinaSeri, PaoloGorisek, ŽeljkoSerier-Brault, HélèneGorji, Nima E.Serikov, ArkadyGórka, JacekSerpooshan, VahidGornakov, VladimirSerra, AngelsGornostyrev, YuriSerra, RogerGorodkiewicz, EwaSerrà, AlbertGorseta, KristinaSerrancolí, GilGorski, ŁukaszSerrano-Aroca, ÁngelGórski, MarcinSethmann, IngoGorunov, Andrey IgorevichŠetina Batič, BarbaraGoryacheva, IrinaSgouros, AristotelisGoryaeva, AlexandraSgroi, Mauro FrancescoGorzelańczyk, TomaszSguazzo, CarmenGossage, R. A.Shaaban, Ibrahim G.Goswami, SubhadipShabalin, IvanGoswami, TarunShaban, AbdulGoto, YosukeShaburova, NataliyaGottschalk, AxelShadloo, Mostafa SafdariGoudoulas, ThomasShafique, UmarGoula, Maria A.Shah, Furqan A.Goulas, AthanasiosShaha, SugribGousheguir, Seyed MohammadShaha, Sugrib KumarGovan, JosephShahmoradi, MahdiGovind Rao, Vishal GovindShahnewaz, MdGoyal, ShwetaShakor, PshtiwanGraber, LukasShala, AhmetGrabham, NeilShalaby, MohamedGrabon, W.A.Shalchi Amirkhiz, BabakGrabowski, GrzegorzShallcross, SamGraciani, EnriqueShamonin, MikhailGraeve, Olivia A.Shams, S. FatemehGräf, StephanShan, XuechuanGragnani, Gian LuigiShang, JunlongGrajcar, AdamShankar, EswarGrajek, HenrykShanmukaraj, DevarajGranado, M. DoloresShao, LuGranda, MarcosShao, SongdongGrande, FedoraShao, YongboGrande Tovar, Carlos DavidShariat, Bashir SamsamGräning, TimSharkeev, YuriiGrant, Gerald T.Sharker, Shazid Md.Grantham, MichaelSharma, SaumyaGrasley, Zachary C.Sharma, Sunil K.Grässel, SusanneSharma, Gyani ShankarGrasso, MarzioSharma, KumariGrattieri, MatteoSharma, YogeshGrau, GerdSharma, Sandan KumarGravel, EdmondSharma, AshutoshGray, VeronicaSharma, PriyankaGraziani, AndreaSharma, Sanjeev K.Graziani, GabrielaSharma, AnuragGrazzini, AlessandroSharma, AmitGrebel, HaimShaulsky, EvyatarGrebenisan, GavrilShavandi, AminGrecco, GiuseppeShayesteh Moghaddam, NargesGreco, AntonioShcherbakov, Maxim R.Grecu, EugeniaShchetinin, IgorGreen, DavidShehu, RafaelGreenman, JohnSheidaei, AzadehGregg, DanielShemelya, CoreyGregori, AlbertoShen, YiouGribniak, ViktorShen, EricGric, TatjanaShen, KelvinGrigsby, Christopher L.Shen, ZhiqiangGrilc, MihaShenashen, Mohamed A.Grilec, KresimirShenderovich, Ilya G.Grill, RomanShepherd, Jennifer H.Grima, Joseph N.Sher, PraveenGrippaudo, CristinaShevtsov, SergeyGrischke, JasminShi, XijunGrivel, Jean-ClaudeShi, ChenGrizzuti, NinoShi, KaiyuanGroch, PawełShi, YongqianGroen, PimShi, QiweiGroh, SebastienShi, ZengqianGromada, MagdalenaShi, ZhenguoGronchi, PaoloShibasaki, MasakatsuGrondin, FrédéricShie, Ming-YouGronostajski, ZbigniewSHIGA, KeijiGrossi, MarcoShih, Ming-ChengGrugeon, SylvieShilko, Evgeny ViktorovichGrum, JanezShim, JiwookGrumezescu, Alexandru MihaiShimada, KeitaGrunwald, TimShimamura, YoshinobuGrupp, Thomas M.Shimizu, KazuoGruttadauria, AndreaShimizu, IchiroGrützmacher, Philipp G.Shimo, TsuyoshiGryc, KarelShimoi, NorihiroGryshkov, OleksandrShimojo, MasayukiGrząbka-Zasadzińska, AleksandraShimosako, NaokiGrzelak, KrzysztofShin, Weon HoGrzelczyk, DariuszShin, Yoon HyukGrzyb, JoannaShin, Su-JungGu, YuweiShin, Crystal S.Gu, WentingShin, Jae SupGu, JunweiShin, Kyung JoonGu, ChunjuShin, MikyungGuarino, StefanoShin, YooseokGuazzelli, LorenzoShirai, KouunGuazzelli, ElisaShiraiwa, TakayukiGubanska, IgaShirasu, KeiichiGubernat, AgnieszkaShirayama, SakaeGucci, FrancescoShirokov, Vladimir B.Gudiminchi, RamaShirvanimoghaddam, KamyarGudmundsson, Jon TomasShiryayev, OlegGüemes, AlfredoShishehbor, MehdiGueorguiev, GueorguiShishkin, AndreiGuerra Rosa, LuisShishkovsky, IgorGuerrero-Vaca, GuillermoShiue, Ren-KaeGuglielmino, EugenioShnyder, SteveGui, ZheShoffstall, AndrewGuijt, RosanneShojaei, Mostafa FaghihGuilhot, DenisShokouhimehr, MohammadrezaGuilleminot, JohannShong, BonggeunGuisbiers, GregoryShoppert, AndreiGuizani, LotfiShrivastav, Gaurav P.Gumfekar, SarangShtepliuk, IvanGunasegaram, Dayalan R.Shtrepi, LouenaGunasekara, ChamilaShunmugasamy, Vasanth C.Gunawidjaja, RayShvidchenko, Aleksandr V.Gunderov, Dmitriy V.Shvydka, DianaGunkelmann, NinaShyha, IslamGun'ko, YuriiSi, GuangyuanGünther, KarstenŠiaučiūnas, RaimundasGuo, ShengSičáková, AlenaGuo, SiqiSiddiq, M. AmirGuo, DezhouSidebottom, MarkGuo, HaiqingSidorenko, AlexanderGuo, XiaofeiSierra, TeresaGuo, QiuquanSievänen, ElinaGuo, XiaofengSignore, GiovanniGuo, JiangSikdar, ShirsenduGuo, DandanSikder, PrabahaGupta, RishiSikirzhytski, VitaliGupta, SanjuSikora, PawełGupta, Ram K.Sikora, JanuszGupta, ChiragSikorski, WojciechGurau, GheorgheSilaghi, Ciprian N.Gurau, CarmelaŠiler, PavelGurevich, Vsevolod V.Silikas, NickGurikov, PavelSilina, Yuliya E.Gurung, AshimSiliqi, DritanGurzawska, KatarzynaSiller, HectorGurzhiy, Vladislav V.Silva, FranciscoGusella, VittorioSilva, Rui VascoGutierrez, Miguél ÁngelSilva, Francisco J. G.Gutiérrez-Capitán, ManuelSilva, António SantosGuzman, MarceloSilva, Teresa Moura E.Guzmán, EduardoSilva, Hugo M.R.D.Gwalani, BharatSilva, AbilioGyurkó, ZoltánSilva, M. BeatrizHa, Heon-YoungSilva, André R. R.Ha, William N.Silva Cardoso Brandão, Ana TeresaHaberfehlner, GeorgSilvan, Miguel MansoHabib, Md SelimSilvello, AlessioHabib, AnowarulSimanjuntak, Firman MangasaHabrat, WitoldSimari, CataldoHachemi, ImaneSimha Martynkova, GrazynaHackemann, StefanSimitzi, CharaHadad, YakirSimka, WojciechHaddag, BadisSimocko, ChesterHadjiargyrou, MichaelSimoen, EddyHadjikakou, Sotiris K.Simoes, VascoHadzima-Nyarko, MarijanaSimões, SóniaHaegel, Franz-HubertSimões, RogérioHafezi, Mohammad HadiSimon, Jaan WillemHaghbin, NaserSimon, CarstensHaghshenas, HamzehSimončič, BarbaraHahn, Jae RyangSimonds, BrianHai, Faisal I.Simonelli, MarcoHai, TranSimonenko, E. P.Haidemenopoulos, Gregory N.Simonutti, RobertoHaifeng, LiuSimserides, ConstantinosHajimohammadi, AilarSimunovic, KaticaHajizadeh, SolmazSimurda, TomasHajtó, DánielSinescu, CosminHakimi, OrlySing, Swee LeongHalilovič, MiroslavSingh, SubhashHall, ChristopherSingh, Ajay VikramHall, SteveSingh, Jitendra KumarHallil, HamidaSingh, JitendraHalmešová, KristýnaSingh, AshishHamad, KotibaSingh, MadhavHamada, ShigeruSingh, GurwinderHamdana, GerrySingh, JaiHamdaoui, OnsSingh, RanjeetHamilton, CarterSinha, Sudarson SekharHamon, MorganSinha, JasmineHampl, AlešSinha, SudarsonHampton, MichaelSinha Ray, SumitHampton, JenniferSinjab, KhaledHamza, OmarSinjari, BrunaHan, Dong-WookSinn, GerhardHan, Gill SangSiqueira, NatalyHan, PingpingSiracusa, ValentinaHan, MeikangSirivolu, DushyanthHan, Soo DeokSirkeli, Vadim P.Han, Dong-PyoSiska, FilipHan, YiweiSismanis, PanagiotisHan, Hyoung-SuSitko, DorotaHanaor, DorianSitnikova, ElenaHandge, Ulrich A.Šittner, PetrHandoko, WilsonSivasubramanian, KathyayiniHandzlik, PiotrSiwak, PiotrHaneke, EckartSiwiec, TadeuszHang, Da-RenSiwińska-Ciesielczyk, KatarzynaHangai, YoshihikoSiwiński, JarosławHanif, AsadSjödahl, MikaelHaniu, HisaoSkachkov, DmitryHänninen, HannuSkaf, MartaHano, ChristopheSkafte, Theis LøyeHansen, Klaus SchüttSkapin, Sreco D.Hanus, Jean LucSkarżyński, ŁukaszHanyková, LenkaSkellam, ElizabethHao, YongSkibicki, DariuszHaque, Md Minhaz-UlSkoda, DavidHaque, RezwanulSkórczewska, KatarzynaHara, KenjiSkordaris, GiorgosHaraldsen, Jason T.Skorik, Yury A.Harbulakova, Vlasta OndrejkaSkotak, MaciejHarding, IanSkouras, AthanasiosHardy, JohnSkowroński, ŁukaszHardy, John GeorgeSkripalenko, MichaelHarhash, MohamedSkripkiunas, GintautasHarik, IssamSkulski, RyszardHariri-Ardebili, M. AminSkwarek, EwaHarizi, WalidSłaby, EwaHarja, MariaŠlapáková, MichaelaHarnois, MaximeSlavov, StoyanHarp, Joel M.Slebodnick, CarlaHarrison, RobertSlepicka, PetrHärtel, SebastianSlepski, PawelHartenbach, IngoSlifka, Andrew J.Hartmaier, AlexanderSliseris, JanisHartman, Matthew C.T.Sližytė, DanutėHartmann, MichaelSlobodyan, MikhailHartwig, K.T.Słodki, BogdanHasan, AbsharSlokar Benić, LjerkaHasanian, MostafaSłomkowski, StanislawHasanzadeh Kafshgari, MortezaŚlosarczyk, AgnieszkaHasegawa, MakotoSłowik, MieczysławHashemian, LeilaŚlusarczyk, ŁukaszHasheminejad, NavidSmaga, MarekHassanin, HanySmallenburg, FrankHatada, RurikoSmarzewski, PiotrHatakeyama, KazutoSmått, Jan-HenrikHattar, KhalidSmet, MarioHattori, TomokazuSmet, PhilippeHattori, ToshioSmetana, VolodymyrHattori, Azusa N.Smialek, James L.Haubrich, JanSmirnov, Ivan V.Haubruck, PatrickSmith, Leonard B.Haudin, Jean-MarcSmith, JamesHaugen, Håvard JSmolin, IgorHaugen, Astri BjørnetunSmolka, PetrHaušild, PetrSnegir, Sergii V.Hausmann, JoachimŠneideris, ArnoldasHawk, JeffreySnopiński, PrzemysławHayakawa, TohruSnyder, NicoleHayashi, FumitakaSoares, Paula IsabelHayashi, HisashiSoares, Graca M.B.Hayashi, YoheiSobaszek, MichalHayashi, KatsuroSobiesiak, MagdalenaHazar, SelcukSobotova, LydiaHe, DelongSobus, JanHe, YingxinSocha, TomaszHe, WeilueSocha, GrzegorzHe, ShaojianSocha, LadislavHe, ChuanglongSoe, ShweHe, HuijingSofia, DanieleHe, Mo-RigenSohal, SandeepHe, HanSohn, Seok SuHe, JinSohn, Jung WooHe, HongliangSohrabpoor, HamedHe, MingyuSoive, AnthonyHe, ZhouyingSokolov, OlegHe, GuanjieSokolova, Maria P.He, JieSokolovsky, VladimirHe, YongSolaberrieta, EnekoHeadings, LeonSolano, FranciscoHebling, JánosSolano, WilberthHedhammar, MySoldano, CaterinaHedjazi, SamanSoleimani, MajidHee, Ay ChingSoler, MariaHegab, HussienSolis, Rafael R.Hegedűs, CsabaSolís, CeciliaHegmann, EldaSolitro, GiovanniHeift, DominikusSollazzo, GiuseppeHeijungs, ReinoutSologubenko, AllaHeim, FredericSolonin, AlexeyHeise, Herbert MichaelSolsona, BenjamínHejna, AleksanderSoltani, ImanHektor, JohanSoltani, AliHeller, LudekSoltani, AminHellmich, ChristianSoman, RohanHellweg, ThomasSomidin, FloraHelman, JoannaSomireddy, MadhukarHelseth, LarsSommer, NicoleHeltzel, JacobSon, Min-KyuHémadi, MiryanaSong, Jeong-HoonHemanathan, KumarSong, MiaoHempel, MartinSong, JingfengHemraz, Usha D.Song, HengxuHenderson, Luke C.Song, XuanHenniges, UteSong, ShaopinHenningsen, AndersSong, KenanHensel, SebastianSong, ErdongHer, Shiuh ChuanSong, ZhaoningHeräjärvi, HenrikSong, Chang EunHerckes, PierreSong, YooseobHermans, IveSongmene, VictorHermawan, HendraSonnier, RodolpheHermida-Merino, DanielSonsteby, Henrik HovdeHernández, Margarita GonzálezSoomro, MahfoozHernández, Nacú B.Soon, Jong JeongHernández Saz, JesúsSopanen, JussiHernández-Nava, EverthSophocleous, MariosHernández-Negrete, OfeliaSorensen, Carl D.Hernandez-Vazquez, Jesus-MariaSorgato, MarcoHernando, DavidSorrentino, AndreaHerrmann, HeikoSorrentino, LucaHerrnsdorf, JohannesSorrentino, LuigiHerten, MonikaSotiriadis, KonstantinosHesketh, JamesSotiropoulos, SotirisHesp, Simon A.M.Soto, RobertHess, DennisSoto, PaulHetmańczyk, ŁukaszSotome, MasatoHeuken, MichaelSougrati, Moulay TaharHey, JeremiasSoukal, FrantisekHeydari Gharahcheshmeh, MeysamSounthararajah, ArooranHidalgo-Carrillo, JesusSousa, LuísHigashikawa, KoheiSousa, Hélder S.Higuchi, TsunehikoSousa, Ana I.Hikawa, HidemasaSoust-Verdaguer, BernardetteHildreth, BlakeSovány, TamásHill, DanielSovják, RadoslavHill, GrantSowa, MaciejHilloulin, BenoîtSpada, AntoninoHinds, GarethSpagnoli, AndreaHinman, SamuelSpagnuolo, GianricoHinterstein, ManuelSpanopoulos, IoannisHippler, RainerSperanza, DomenicoHirata-Tsuchiya, ShizuSperanzini, EmanuelaHiremath, NitilakshaSpieckermann, FlorianHirohata, MikihitoSpieß, LotharHirota, YuichiroSpina, RobertoHirsch, ThomasSpinelli, GiovanniHisada, KenjiSpingler, BernhardHlaváč, Libor M.Spinsante, SusannaHlaváčová, Irena M.Spišák, EmilHlosta, JakubSpoerk, MartinHnida, KatarzynaSportelli, Maria ChiaraHo, Yi-ChengSpowage, AndrewHo, Ko-ShanSprouster, DavidHo, Chia-cheSrinivasan, BhuvaneshHobosyan, MkhitarSrinivasan, SeshaHoch, ConstantinSrinivasan, GowrishankarHodoroaba, Vasile-DanSriram, RashmiHoff, IngeSrivastava, G.P.Hoffman, JasonStachewicz, UrszulaHoffmann, AndreasStachiv, IvoHoffmann, DanielStahl, UllrichHoffmann-Eifert, SusanneStåhl, Jan-EricHofman-Caris, RobertaStan, MirunaHofmann, MichaelStanciu, Mariana DomnicaHoja, JohannesStanciu, GeorgeHojo, TomohikoStancu, CristinaHokamoto, KazuyukiStanculescu, IoanaHoła, JerzyStaneva, DesislavaHoldengreber, EldadStanko, DavorHolderer, OlafStarikov, SergeiHolec, DavidStarostenkov, MikhailHolldack, KarstenStaruch, MargoHöller, ChristianStaszak, KatarzynaHollerbach, RainerStaszuk, MarcinHolschemacher, KlausStavrinides, Stavros G.Holynska, MalgorzataStavropoulos, PanagiotisHomaeigohar, ShahinStavroulakis, Georgios E.Honda, MasakiStawarz, MarcinHong, TaoSteensels, RikHong, Sung-GulStefan, RazvanHong, SukjoonStefanidou, MariaHong, MinStefanik, AndrzejHong, YoungkeunStefanou, GeorgeHong, Soon-JikSteffen, RichardHong, Sung HwanSteinberg, SimonHongbing, DengStelescu, Maria DanielaHonus, StanislavStender, MichaelHooi Cho, ByoungStensen, Jan AgeHooman, AmidStenzel, VolkmarHöppel, Heinz WernerStepanov, NikitaHorchidan, NadejdaStephens, MatthewHorel, ÁgotaStepien, AnnaHorhat, Florin GeorgeStepnik, MaciejHorodek, PawełStępniowski, WojciechHorovistiz, Ana LúciaStergioudi, FaniHorowitz, Robert A.Steriotis, TheodoreHorrocks, RichardStern, AdinHorszczaruk, ElżbietaSterzyński, TomaszHorvat, BarbaraStine, Keith J.Horvath, TomasStirner, ThomasHorváth, OttóStjepanović, MarijaHorwat, DavidStoch, PawełHosein, YaraStochino, FlavioHosemann, PeterStoffels, Shelley M.Hoshian, SashaStoica, Grigoreta M.Hosier, Ian LStojanović, SanjaHosokawa, AtsushiStojcevski, FilipHossain, MokarramStojmenović, MarijaHossain, Shahriar A.Stokowa-Sołtys, KamilaHossain, M.NurStolarczyk, AgnieszkaHossain, MohammadStoleru, ElenaHosseini, Vahid A.Stommel, MarkusHosseinpoor, MasoudStone, KariHosseinpour, SamanStopić, SrećkoHosseinpourpia, RezaStorchak, MichaelHossfeld, MaxStorti, EnricoHostaš, JiříStoulil, JanHousecroft, CatherineStøvring, Jan LuxhøjHowaniec, NataliaStoyan, DietrichHoward, IanStraka, L’uboslavHowell, BobStrąkowska, AnnaHowes, PhilipStrankowski, MichalHoyos, PilarStráský, JosefHozumi, KentaroStratakis, EmmanuelHribernik, SilvoStraumal, Boris B.Hristov, IlianStraže, AlešHryniewicz, TadeuszStrečka, JozefHrynyk, TrevorStręk, TomaszHsia, Shao-YiStrelniker, YakovHsiao, H.L.Streubel, RobertHsieh, Chien-KuoStride, John ArronHsu, Cheng-HsunStrieder, EmanuelHsu, Yung-JungStringer, Jonathan EdwardHsu, Fu-YinStrozzi, MatteoHsu, Tuan-TiStrube, JochenHsu, Chia ChenStruzikiewicz, GrzegorzHsu, Jin-CherngStryszewska, TeresaHsu, Wei-HsuanStrzałkowski, KarolHsueh, Yi-HuangStrzemiecka, BeataHu, YangStuart, BryanHu, JunStucchi, MartaHu, GuopingStupakov, AlexandrHu, JingjieStuparu, MihaielaHu, ChuanlinSturini, MichelaHu, Yong-JieStylianakis, Minas M.Hu, ShanSu, YingchaoHua Shi, YongSu, Tzu-SenHuan, XunSu, An-ChungHuang, Heng LiSu, Pi-GueyHuang, QijinSu, ChangshengHuang, Chia-HuaSu, ZhouchengHuang, HuiSu, Chiung-WuHuang, Chih-ChingSuárez, FernandoHuang, WeixinSuárez, RafaelHuang, ChaoboSuárez, IsaacHuang, Chun-YingSubotić, VanjaHuang, Chih-LingSubramani, ThiyaguHuang, ChenyuSuchanicz, JanHuang, LiangSucharda, OldrichHuang, Kuo-ChengSuchorzewski, JanHuang, Han HsiangSudarsanam, PutlaHuang, Heng-LiSudhakar, VadirajaHuang, JingfengŠugár, PeterHuang, HuayuSugawa, KosukeHuang, HantaoSugisaki, KenjiHuang, Shyh-ChourSugiura, YukiHuber, David L.Suh, Jin-YooHuber, ChristianSuh, DongseokHubert, OlivierSukegawa, ShintaroHübner, ChristianSuLing, ZhaoHudak, RadovanSulitka, MatejHuelva, Marta MolinaSullivan, TimothyHughes-Riley, TheodoreSumelka, WojciechHuh, JungwonSummerscales, JohnHuh, SeongSun, HaoHuh, Yun SukSun, ChengqiHulme-Smith, Christopher NeilSun, ShaolongHumad, Abeer M.Sun, YongHumar, MihaSun, XiuxuanHumphries, Jonathan D.Sun, JiaminHumpolicek, PetrSun, HongyuHung, Pui-yanSun, LemingHung, Tsung PinSun, MengHung, WayneSun, XiangchengHuo, SuguoSun, ShijingHuo, QinghuanSun, YongleHuot, JacquesSunatsuki, YukinariHur, DohaengSundaram, PaulHurley, MikeSunna, AnwarHurschler, ChristofSuñol, Joan-JosepHusanu, ElenaSurace, RossellaHusev, OleksandrSuraneni, PrannoyHussein, HusamSurleva, AndrianaHussein, RamySurmenev, Roman A.Hüter, ClaasSurreddi, Kumar BabuHütter, GeralfSurženkov, AndreiHuu Nguyen, TrungSusca, TizianaHuynh, Thanh-CanhSutariya, VijaykumarHvizdoš, PavolSutka, AndrisHwang, YongsungSutradhar, ManasHwang, Chi-SunSu-Wen, HsuHwang, Chao-LungSuzuki, YasushiIacobellis, VincentSuzuki, NorihiroIanchis, RalucaSuzuki, Jon B.Iannazzo, DanielaSvavarsson, Halldor GudfinnurIatsunskyi, Igor RSveda, MariaIbrahim, HamdySvenningsen, GauteIbrahim, AhmedSvensson, Ann MariIbrahim, ToniSverdlov, ViktorIchikawa, YoSvetlichnyi, Valery A.Iconaru, Simona LilianaSvintsov, Alexander P.Ida, ShoheiSvirshchevskaya, ElenaIdczak, RafałSvoboda, JiriIelciu, IrinaSwadźba, RadosławIezzi, GianlucaSwadzba-Kwasny, MałgorzataIfelebuegu, AugustineSwiatkowska, ZanetaIglesias, EmiliaŚwiderski, WaldemarIgnaszak, AnnaŚwięch, ŁukaszIgnatova, TetyanaŚwiercz, RafałIgnés-Mullol, JordiŚwierczyńska, AleksandraIjima, HiroyukiŚwiętosławski, MichałIkeda, AtsushiŚwit, GrzegorzIkesue, AkioSypien, AnnaIkram, AwaisSyrek, KarolinaIlan, ShalishSzabelski, JakubIlanchezhiyan, PugazhendiSzala, MiroslawIlcikova, MarketaSzala, MateuszIliev, BoyanSzaller, ZsuzsannaIljkić, DarioSzałowski, KarolIllana, Irene Del SolSzalwinski, ChrisIllangakoon, U.ErankaSzczepanik, BeataIllés, BalázsSzczepanski, CarolineIllescas Montes, RebecaSzczes, AlexandraIllia, DobrydenSzekalska, MartaIlnicka, AnnaSzekely, GyorgyImperatore, StefaniaSzeląg, MaciejInada, RyojiSzeleszczuk, ŁukaszInam, Muhammad AliSzepvolgyi, JanosIndrawan, NatariantoSzewczyk, RomanInfantes-Molina, AntoniaSzilagyi, HenrietteIngram, ConradSzilágyi, IstvanIngrassia, TommasoSzkliniarz, AgnieszkaIno, KosukeSzkodo, MarekInomata, KatsuhiroSzlancsik, AttilaInozemtcev, AleksandrSzolnoki, BeátaIoana, AdrianSzopa, KrzysztofIon, Rodica-MarianaSzostak, MarekIon-Ebrasu, DanielaSzpikowska-Sroka, BarbaraIonescu, Mihaela IleanaSzuber, JacekIonita, PetreSzulc, PiotrIop, LauraSzumełda, TomaszIordache, FlorinSzutkowska, MagdalenaIordanskii, Alexey L.Szuwarzyński, MichałIorio, Ronald M.Szwajka, KrzysztofIpus, Jhon J.Szybinski, BogdanIqbal, ZafarSzybowicz, MiroslawIrani, MissamSzymański, WojciechIrena, KratochvílováSzymczyk, MagdalenaIrene Urbieta Quiroga, AnaSzymczyk, AnnaIrez, Alaeddin BurakSzyszka, JerzyIrimiciuc, Stefan-AndreiSzyszlak-Bargłowicz, JoannaIrvine, Scott A.Ta, HangIsaev, AbdulgalimTabacaru, AurelIsakov, DmitryTabacchi, GloriaIsanaka, Sriram PraneethTabakovic, AmirIsbrandt, MarekTabaran, FlaviuIshikawa, YasuakiTabatabaei, AlirezaIshikawa, SatoshiTabei, AliIshikawa, ToshihiroTabish, TanveerIshikawa, RyoTaboada-Serrano, PatriciaISIK, MuratTada, NaoyaIskierko, ZofiaTada, RuiIskratsch, ThomasTaddei, PaolaIslam, MohammadTadesse, MelkieIslam, Md RashadulTadokoro, ChiharuIsobe, NoriyukiTadyszak, KrzysztofIsola, GaetanoTae-Jun, HaIsopescu, RalucaTaflove, AllenIspas, AdrianaTafraoui, AhmedIstrate, OanaTaghizadeh, RuhollahIstrate, Irina AuraTaglietti, Angelo MariaIto, MikioTahami, Seyed AmidIto, TakeruTahara, HironobuIto, KazuhiroTaheri, AbbasItoh, TamitakeTaheri, HosseinItri, AngeloTaheri, ShimaIuliana, HudisteanuTaheri, RezaIván, BélaTahmoorian, FarzanehIvančević, DarkoTai, Bruce L.Ivanišević, AnaTailhades, PhilippeIvanova, BojidarkaTakada, TomoyaIwamoto, TakeshiTakada, AkihikoIwański, MarekTakaffoli, MahdiIyer, AbishekTakagaki, AtsushiIzquierdo, JavierTakahashi, KeisukeJaafar, MiriamTakahashi, KojiJabraoui, HichamTakahashi, YasuoJacek, ŻmojdaTakakado, AkiraJackson, MarkTakamizawa, ToshikiJackson, MichaelTakano, HajimeJacquet, PhilippeTakarics, BélaJacukowicz-Sobala, IrenaTakashima, ToshihiroJaczewski, MariuszTakeda, YasunoriJadczak, Joanna N.Takegahara, NorikoJadwicienczak, WojciechTakoudis, Christos G.Jaganathan, ArunTakura, TomoyukiJaggessar, AlkaTalaat, AhmedJagtap, SujitTalhi, OualidJahangir, IfatTalimian, AliJahangiri Koohbanani, BehnamTallapally, VenkateshamJahn, MarcusTallarico, MarcoJahromi, HosseinȚălu, ȘtefanJain, AnkurTalukdar, SudipJain, GauravTamadon, AbbasJakić, MićeTamarin, OllivierJakieła, SławomirTamás, MankovitsJakšić, OlgaTamas-Gavrea, Daniela-RoxanaJakubas, AdamTamulevičienė, AstaJakubczak, PatrykTan, XipengJakubczyk, EwaTan, CaiwangJakubowicz, JarosławTan, XianjunJalali, MandanaTan, Kui-ThongJamalabadi, Mohammad Yaghoub A.Tan, HongboJames, SagilTanaka, MasatoJammalamadaka, UdayabhanuTanaka, KatsushiJamrozik, WojciechTanaka, KojiJamshidi, ReihanehTang, MingJana, AtanuTang, ChaolongJanas, DawidTang, Chao-WeiJanczarek, MarcinTang, Yunchao TangJanda, TomasTang, HouliangJaneček, MilošTang, WilkinJänes, AlarTang, XiaochaoJaneta, MateuszTang, Cheng-MingJang, JinahTang, LongtengJang, Sung-HwanTanjil, MdRubayat-E.Jang, Jeong GookTanner, EdenJang, SoohwanTanno, TakenoriJang, Seung-YupTański, TomaszJang, JaewonTanyeri, MelikhanJanga, Srikanth ReddyTao, ChengchengJanicki, DamianTao, KaiJanikowska, GrażynaTapoglou, NikolaosJanka, OliverTappa, KarthikJankauskaite, VirginijaTarallo, OresteJankovic, JasnaŢǎranu, NicolaeJanovec, JozefTarasiuk, W.Janovszky, DoraTarasov, Sergei YuJanowski, MiroslawTarasov, IvanJanus, RafałTardy, BlaiseJanus, MagdalenaTarfaoui, MostaphaJaques, BrianTaruta, SeiichiJarosz, TomaszTaskinen, PekkaJasielec, Jerzy J.Tasolamprou, AnnaJasiewicz, MarcinTatar, JovanJasim, Ali A.Tataranni, PiergiorgioJaskuła, PiotrTatarko, PeterJaskulski, RomanTatsumi, HiroakiJaśniok, MariuszTatsumi, YukiJastrzab, RenataTattikota, Sudhir GopalJatale, AnchalTavakoli, JavadJauregui, CatherineTavangarian, FariborzJavaherdashti, RezaTavanti, FrancescoJaved, HassanTavares, S.M.O.Javier Baeza, FranciscoTavares, PedroJayasekera, ThushariTawfik, Sherif AbdulkaderJazaei, RobabehTawil, Bill J.Jędrzejczyk, Roman J.Tay, Daniel YiweiJędrzejewska, AgnieszkaTayeb, Ali H.Jedynak, KatarzynaTayel, AmrJeffs, SpencerTaylor, Thomas J.Jégou, SébastienTcherdyntsev, Victor V.Jelliss, PaulTealdi, CristinaJelonek, KatarzynaTebaldo, VincenzoJenab, ArashTedim, JoaoJencova, VeraTeghil, RobertoJeng, Jiiang-HueiTehrani, MehranJeng, Ming-JerTeichert, NiclasJenko, MonikaTeimourian, AmirJennings, Abby R.Teixeira, Erica C.Jenuš, PetraTejchman, JacekJeon, Ju-WonTelang, AbhishekJeong, Dae-YongTelegdi, JuditJeong, Hyun-damTeleki, AlexandraJeong, Nak CheonTenchov, BorisJeong, Hyung MoTeng, ChongJeong, YeonungTenorio-Alfonso, AdriánJeong, Keun-YeongTenza-Abril, Antonio JoséJeong, Hoon EuiTeodorescu, FlorinaJeong, Chang KyuTeoh, Yu-YaoJeremiáš, MichalTeramoto, YoshikuniJermakowicz-Bartkowiak, DorotaTerčelj, MilanJerman, MilošTercjak, AgnieszkaJesson, DavidTeresa, MangialardiJessu, Kashi VishwanathTerner, MathieuJezierski, JanTessore, FrancescaJeziorska, ReginaTesta, GabrielJezowski, PawelTestani, ClaudioJha, Ramesh K.Thackray, RichardJi, VincentThai, Duc-KienJi, ShouxunThai Duy, LeJia, HailongThakur, Vijay KumarJia, MingtuThang, Tran QuyetJia, XiaodongThanigaimani, ShivshankarJia, YunlongThapa, DineshJia, YandongThapliyal, VivekJia, ZhenyuanTheodorakis, PanagiotisJia, YuTheres Baby, TessyJiang, ShaohuaThiebault, ThomasJiang, ZhuangDeThinon, EmmanuelleJiang, JunThives, LiseaneJiang, WenThiyagarajan, Jothi SaravananJiang, PingThomas, PhilippeJiang, ShuyongThompson, RobJiang, ZiwenThomsen, Jesper S.Jiang, XuchuanThrivikraman, GreeshmaJiang, WenbinThuault, AnthonyJiang, RuinianThuvander, MattiasJiao, YuboTiainen, HannaJiao, XuechenTian, YanzhongJicsinszky, LászlóTian, HeJiménez, AmaiaTian, LiangJimenez Alonso, Javier FernandoTicha, HelenaJimenez-Rivero, AnaTien, Chuen-LinJimenez-Villacorta, FelixTikhonov, VladimirJin, XiaoliangTimashev, PeterJin, ShengliTimmel, KlausJin, Chul KyuTimothy, Jithender J.Jin, QianruTing, Jeffrey M.Jin, JianyongTiwari, Kumar AnubhavJin, GuofanTiwari, AvinashJin, JingTkach, AlexanderJin, Hyeong MinTkachenko, NikolaiJindo, KeijiTo, PeterJinno, YoheiTobaldi, David MariaJirková, HanaTocci, MarialauraJishkariani, DavitTodaro, LuigiJo, SungkwonTodros, SilviaJo, Young-minTofan, LaviniaJoe, PayerTofangchi, AlirezaJohannes, KieferTofel, PavelJohansson, ChristerToffoli, AndreaJohnson, Oliver K.Tojo, TomohiroJohnson, DavidTokarska, MagdalenaJohnson, ScooterTokovyy, YuriyJohnsson, MatsTokumoto, YukiJokić, DraženTole, IldaJoly-Duhamel, ChristineTolkou, AthanasiaJomaa, WalidTölli, HannaJones, FrancaTolvanen, JarkkoJones, HywelToma, Ionut-OvidiuJoo, JonghoonTomanek, BoguslawJoo, Soo-HyunTomastik, JanJoos, JonasTomasz, DurejkoJopek, HubertTomasz, MoscickiJordana, FabienneTomczak, JanuszJorge Evangelista, Ana CatarinaTomczyk, KrzysztofJorquera-Lucerga, Juan JoséTomer, Vijay K.Joseph, OlufunmilayoTomków, JacekJoseph, StephenTomoaki, KarakiJoseph, J. MarcinkoTompos, AndrásJoseph, FrancesTomšič, BrigitaJoshi, NiravkumarToncheva, AntoniyaJoshi, SurajTonelli, MonicaJothi, Palani RajaTong, LinyueJozwiak, StanislawTong, Van-CanhJozwiak-Niedzwiedzka, DariaTopolář, LiborJozwik, PawelToprakci, Hatice Aylin KarahanJubeli, EmileTorgal, F. PachecoJucius, DaliusTorikai, HiroyukiJuergens, PhilippTornabene, FrancescoJuhna, TalisToro, Roberta G.Julien, Christian M.Török, TamásJun, YoungchulTorop, JannoJun, ByungMoonTorreggiani, ElenaJun, Bong-HyunTorres Hernández, YadirJunak, JozefTorres Salcedo, AlexiaJung, YongwonTorres Vaamonde, José EnriqueJung, DongWonTorres-Lagares, DanielJung, Yeon-GilTorzewski, JanuszJung, SeunhoTosaka, MasatoshiJung, Hyun-DoTosello, GuidoJung, DaeseungTost, Ramón MorenoJung, HaesungToth, LaszloJunpeng, LuTóth-Bodrogi, EditJuodkazis, SauliusTotu, EugeniaJurado Sánchez, BeatrizToudert, JohannJuraic, KrunoslavToumpanaki, EleniJurči, PeterTracz, TomaszJurczyk, MieczyslawTraini, ToninoJurdziak, LeszekTrajkovski, BrankoJürgen, BärTrakakis, GeorgeK. Sharma, SunilTran, JonathanKaabouch, NaimaTran, FabienKaal, WilliamTran, Thien ToanKaars, JonnyTran, Van-ThaiKabiri, Ameri ShidehTran, Lam Thi NgocKaci, AbdelhakTranquillo, ElisabettaKaczmarczyk, JarosławTrauth, AnnaKaczmarek, BeataTraven, KatjaKaczmarek, Anna M.Traxel, KellenKaczmarek, Slawomir M.Treideris, MariusKaczmarek, HalinaTrejbal, JanKaczmarek, Małgorzata T.Trematerra, AmeliaKaczyński, PawełTretiakov, KonstantinKada, SitaramaTreutler, KaiKadela, MartaTreviño, Roque BorinagaKadela-Tomanek, MonikaTri Phung, QuocKadi, NawarTriantou, MariannaKadokawa, Jun-ichiTridello, AndreaKadumudi, Firoz BabuTrifol, JonKafarski, PawelTrindade, RicardoKafexhiu, FevziTrindle, CarlKafi, AbdullahTrinh, ThuatKageshima, YosukeTripathi, Kumud MalikaKahle, EckhardTriplett, R. GilbertKahler, BillTritto, IncoronataKairytė, AgnėTrivedi, RahulKaisarly, DaliaTrochu, FrançoisKajitani, TakashiTroedhan, AngeloKalacska, SzilviaTroiani, EnricoKalácska, GáborTrojanova, ZuzankaKalamees, TargoTrosien, SimonKalandyk, BarbaraTrotta, GianlucaKalatehjari, RoohollahTrotta, FrancescoKaleta-Jurowska, AlinaTrovalusci, PatriziaKalinkin, AlexanderTrueba, LuisKalinowski, MaciejTrukhanov, AlexeiKaliva, MariaTrukhanov, Sergei ValentinovichKaliya Perumal Veerapandian, SavitaTrunec, MartinKalkowski, Michał K.Trunec, DavidKalligeros, Stamatis SpyridonTruong, Vi-KhanhKallitsis, JoannisTruskey, George A.Kalló, GergőTrutalli, DavideKalogiannis, Konstantinos G.Trybuła, Marcela E.Kalogirou, OrestisTrybuła, ZbigniewKaloni, Thaneshwor P.Trzepiecinski, TomaszKaluža, LuděkTsai, Ang-ChenKalyana Sundaram, Ramalingam VenkatTsai, Dai-ShyangKamali, SaeedTsai, Meng-HsiuKamanina, Natalia V.Tsai, Chen-YenKamat, AmarTsamatsoulis, DimitrisKamatham, NareshbabuTsang, Hing-HoKamdem, DonatienTsangouri, EleniKamel, KarolTsantilis, LuciaKameoka, JunTsao, Lung-ChuanKameyama, AtsushiTsao, Chung ChenKamiguchi, SatoshiTsaturyan, ArshakKamińska, GabrielaTsau, Chun-HueiKamiński, MarcinTsay, CHien-YieKamiya, OsamuTseluikin, VitalyKampmann, RaphaelTseng, Yu-ChuanKanakubo, ToshiyukiTseng, Wei-LungKanari, NdueTsibidis, GeorgeKanda, KazuhiroTsioulou, OuraniaKandhasamy, SathiyarajTsirelson, Vladimir G.Kaneco, SatoshiTsoi, James Kit-honKaneko, KenjiTsompanakis, YiannisKanematsu, HideyukiTsuchiya, AkiraKang, JidongTsuji, YasuhideKang, Sung-HoonTsuji, HidetoKang, XinTsvetanov, ChristoKang, Dong-WonTsvetkov, NikolaiKang, SangminTubaro, CristinaKang, Chung GilTucho, Wakshum M.Kang, Jae-YoonTuci, GiuliaKang, HyoTucker, JulieKang, Young-JongTudorache, FlorinKang, Phil HyunTukhbatullin, AdisKang, JinwuTulliani, Jean MarcKang, WeiTumarkin, AndreyKania, TomaszTumuluri, UmaKania, HenrykTung, Tran ThanhKanicky, VictorTung, Nguyen-DangKanno, TakahiroTuominen, JariKano, JunyaTuovinen, TeroKantartzis, Nikolaos V.Turlo, VladyslavKanyong, ProsperTurner, RichardKanzaki, HiroyukiTuro, DiegoKao, Tsung ShengTuruallo, GidionKapcia, Konrad JerzyTuti, SimonettaKapetanakis, MyronTuyan, MuratKaplan, AndreyTuz, LechosławKapłonek, WojciechTverdokhlebov, SergeiKappagantula, KeertiTylczak, JosephKapsalis, Vasilios C.Tyliszczak, BożenaKara De Maeijer, PatriciaTylkowski, BartoszKarabchevsky, AlinaTzanetakis, GiorgosKarabinis, Athanasios I.Tzetzis, DimitriosKarakawa, MakotoTziavos, NikolaosKaraolia, PopiTzounis, LazarosKarapanagiotis, IoannisUbertini, FilippoKarayannis, ChrisUdalov, OlegKarayiannis, NikosUdroiu, RazvanKarbalaei Baba, AlirezaUeji, RintaroKarbownik, IwonaUemori, TakeshiKarczewski, KrzysztofUeshima, NobufumiKareiva, AivarasUflyand, IgorKarfoul, M.KamalUgrina, MarinKarim, NazmulUgues, DanieleKarimi, Mohammad M.Ukar, EnekoKarimov, DenisUklejewski, RyszardKariz, MirkoUkrainczyk, NevenKar­jalainen, ErnoUlewicz, RobertKarkalos, Nikolaos E.Ulicna, LiviaKarl, Christian WolfgangUllmann, Clemens VinzenzKarlík, MiroslavUllrich, CarstenKarnahl, MichaelUlyashin, Alexander G.Karnati, PriyankaUm, Han-DonKarnati, Konda ReddyUmerska, AnitaKarolus, MalgorzataUnno, MasafumiKarpov, SergeyUnterreiner, Andreas-NeilKarpukhina, NataliaUnterweger, ChristophKartheek, AnekellaUpare, Pravin P.Karvounis, ArtemiosUr, Soon-ChulKarwowska, EwaUrbani, MaxenceKasai, NaoyaUrbanik, MarekKaščák, ĽubošUrbano, Ana MargaridaKaseem, MosabUrbassek, HerbertKashani, HamzehUrbicain, GorkaKashaninejad, NavidUreña, JuliaKashif, MuhammadUrgessa, Girum SolomonKasmi, NejibUrish, Kenneth L.Kasnatscheew, JohannesUshakov, Sergey V.Kataoka, YusukeUshijima, KuniharuKaterski, AtanasUsui, KenjiKatin, Konstantin P.Utton, ClaireKatkov, Vsevolod L.Utu, Ion-DragosKattamuri, PadmanabhaUykur, EceKatunin, AndrzejUznanski, PawelKatz, Jonathan I.Uzunova, Ellie L.Katz-Demyanetz, AlexanderVaclav, SeflKatzer, JacekVadahanambi, SridharKaushik, NagendraVág, JánosKavadiya, ShalineeVahab, MohammadKawabata, YuichiroVahabi, HenriKawaguchi, Shin-ichiVaiana, RosolinoKawai, ToshiyukiVaiano, VincenzoKawakami, HiroshiVaikuntanathan, VisakhKawakita, HidetakaVaitkus, SauliusKawalko, JakubVakalova, Tatyana VictorovnaKawamura, IzuruVakros, JohnKawaoka, ShinpeiVaks, VladimirKayumov, AiratValappil, SabeelKazasidis, Marios E.Valeev, DmitryKazda, TomášValente, RoberttKazek-Kęsik, AlicjaValente, JoanaKazys, RymantasValentin, JanKe, Jia-HongValentina, LoprestoKe, XinyuanValentini, RenzoKeckes, JozefValerga Puerta, Ana PilarKędziora, AnnaValipour, MohammadKeller, BrandisValivonis, JuozasKellesarian, SergioVallejo, Javier P.Kelley, StevenVallianatos, FilipposKelly, RobertValsaraj, AmithrajKengelbach-Weigand, AnnikaValvano, StefanoKenzari, SamuelValvo, Paolo S.Keppert, MartinVamanu, EmanuelKerai, LaxmiVan Der Schaaf, JohnKerch, GarryVan Der Sluis, OlafKerman, KaganVan der Zwaag, SybrandKern, MatthiasVan Driessche, AlexanderKern, FrankVan Erps, JurgenKertmen, AhmetVan Herwijnen, Hendrikus W. G.Keskisaari, AnnaVan Humbeeck, JanKetkaew, JittisaVan Rooyen, Isabella J.Ketoja, JukkaVan Toan, NguyenKetov, SergeyVancaeyzeele, CédricKhabaz, FardinVande Vannet, BartKhachatryan, GoharVanetsev, AlexanderKhademi, FaezehossadatVannozzi, LorenzoKhadka, Dhruba B.Vapnik, YevgenyKhairulina, KaterynaVaradwaj, Pradeep R.Khajotia, Sharukh S.Varevac, DamirKhakalo, SergeiVarga, MarkusKhaledialidusti, RasoulVarga, TamasKhaliq, Dr JibranVarga, GyulaKhan, ArafatVarga, MariánKhan, Muhammad AhmedVargas, Oscar AndresKhan, Inayat AliVarma, Rajender S.Khanal, RabiVarone, AlessandraKhandaker, MorshedVarveri, Aikaterini (Katerina)Khang, GilsonVasconcelos, Helena CristinaKhani, ShaghayeghVasco-Olmo, Jose ManuelKhanolkar, GauriVasilatos, CharalamposKhansur, Neamul HayetVasile, Bogdan StefanKhare, KetanVasilevich, A. V.Kharel, ParashuVasilevskaya, ValentinaKharin, ViktorVasilevskiy, KonstantinKharkar, PrathameshVasiljević, JelenaKhasainov, Boris A.Vatin, NikolaiKhatib, JamalVautard, FredericKhatir, SamirVauthier, ChristineKheradmand, NoushaVavilov, VladimirKhmeleva, Marina G.Vaz, Daniela C.Khodadad, DavoodVazquez Martinez, Juan ManuelKhodadadi, MohammadVázquez Valeo, JesúsKhodadadi, Jay M.Vecchiattini, RitaKhokhlov, NikolayVédrine, Jacques C.Khoshnood, AbbasVedyagin, Aleksey A.Khosravi, AliVega Gutierrez, SarathKhovaylo, Vladimir V.Vega-Zamanillo, ÁngelKhrustalyov, AntonVeiga, María-DoloresKhung, Yit LungVekinis, GeorgeKhurshid, ZohaibVelasco, José IgnacioKia, AlaleaVelasco Lopez, Francisco JavierKiani, Amirkianooshvelasco ortega, eugenioKiefer, RudolfVelasco-Vélez, Juan JesúsKiełbus, AndrzejVelea, Marian NicolaeKiełczyński, PiotrVelegzhaninov, Ilya O.Kiersnowska, AgnieszkaVelkavrh, IgorKihara, Shin-ichiVelliyagounder, KabilanKijenska, EwaVelu, RajkumarKijo-Kleczkowska, AgnieszkaVenanzoni, GiuseppeKik, TomaszVendik, IrinaKikhtyanin, OlegVenditti, IoleKikuchi, TatsuyaVenegas, MariaKikuchi, HiroakiVenetis, JohnKim, Su-HyeonVenkata, Kiranmayi AbburiKim, Young SikVenu, AnnamdasKim, Won-TaeVenuti, ValentinaKim, Won KyuVercher Pérez, Rosa FranciscaKim, Heung-SikVerdeja, Luis FelipeKim, Kyung-TaekVereb, ZoltanKim, JaehyunVereshchaka, Alexey A.Kim, Jong WonVergara, DiegoKim, Moon DeockVergara Rodríguez, DiegoKim, HyunseokVerma, PragyaKim, J. M.Verma, RanjitKim, Chan-JungVermeşan, HoraţiuKim, Byoung SuhkVernardou, DimitraKim, Ii TaeVerona, EnricoKim, Jin-BomVerotti, MatteoKim, Bom SooVerre, SalvatoreKim, WangdoVerros, GeorgeKim, Byung-JooVértesy, GáborKim, Chang SupVertruyen, BenedicteKim, JinsooVervaeke, MichaelKim, HyeongjooVerzak, ŽeljkoKim, KyunghoonVescovini, RiccardoKim, Ki-buemVesel, AlenkaKim, Seung-JooVesenjak, MatejKim, DoyoonVeziroglu, SalihKim, Moon-JoVezzù, SimoneKim, TaehoonVicente, Miguel A.Kim, Yoo-jaeVicente, Manuel Sastre DeKim, JaewooVicenzo, AntonelloKim, Moon IlVictoria-Hernandez, JoseKim, SeunghyunVidal, CatarinaKim, Jeong TaeVidal, HilarioKim, Sung Y.Vidal-Álvarez, GabrielKim, Seong SooVidor, Fábio F.Kim, Hyun JaeVieira, ManuelKim, Keun-SooVieira, E. M. F.Kim, Hyo JungVignali, ValeriaKim, Kyung HoVijayaraghavan, VenkateshKim, SunilVijayavenkataraman, SanjairajKim, Keun SuVikraman, DhanasekaranKim, Jae-HongVikulina, AnnaKim, Tae-HyungVilà, AnnaKim, SokVila-Cortavitarte, MartaKim, SangtaeVilar, RuiKim, HyungwooVilas, José LuísKim, Ki SuVilcakova, JarmilaKim, Seong-KyunVilla, ElenaKim, MinkyumVilla, CristianKim, Seong-GonVilla, AlbertoKim, HyungsunVilla, IreneKim, Dong-jooVillalba, JavierKim, Duck-HoVimmrová, AlenaKim, Jung-GuVinai, RaffaeleKim, Yong-RakVinardell, MariaKim, DaehwanVincent, DidierKim, BongjuVincent, ThomasKim, Myung HyunVinci, AntonioKim, Jin-KookVining, Kyle HolmbergKim, Heung-KyuVinnik, DenisKim, SeokminVinogradov, VladimirKim, Soo YoungVinogradov, SergeyKim, Hyeon-CheolVinokurov, SergeyKim, Dai-SikVinuesa, RicardoKim, Dong-HyunVipulanandan, CumaraswamyKim, YounghoonVirlan, ConstantinKim, Eun-jinVirtanen, HeikkiKim, Ha-NeulVisco, AnnamariaKim, JunghwanVisentin, FrancescoKim, JedoVishnyakov, AlekseyKim, Choong-UnVisintin, PhillipKim, Yong-GunViskadourakis, Zacharias A.Kim, Chung-SeokVišniakov, NikolajKim, Kwang-MahnVisvanathan, RayshanKim, Man-HoeViswanathan, VaishakKim, Yu KwonVitale, EnzaKim, Dae SuVithana, ChamindraKim, HyunsungVittorio, OrazioKim, Gue-HyunViviani, MassimoKim, Tae-HoonVivo, PaolaKim, Dae-YoonVizureanu, PetricǎKimata, MasafumiVladescu, AlinaKimoto, TsunenobuVladkova, TodorkaKimura, YoshiharuVlǎdoiu, RodicaKinjo, HidekazuVlaskin, MikhailKinuthia, JohnVlcak, PetrKinzel, CarolinaVo, Duc-ThangKioseoglou, GeorgeVo, TruongKirilov, PlamenVodyankina, OlgaKirschhock, Christine E. A.Vogel, ThomasKiryukhantsev-Korneev, Ph.V.Vogiatzis, GeorgiosKis, Viktoria K.Vogt, ChristianKishore, VipuilVogt, OliverKisielewicz, TomaszVogt, JochenKiss, ImreVoicu, Stefan IoanKitagawa, JiroVoit, KlausKitagawa, DaichiVojtěch, DaliborKitahara, TatsumiVojtova, LucyKitajou, AyukoVoliani, ValerioKitchen, JonathanVolk, WolframKittner, KristinaVolker, KörstgensKityk, IwanVölker, BernhardKiwi, John T.Volkov, VladimirKizek, JánVolkova, OlenaKlapötke, Thomas M.Volkova, Elena G.Klein, ThomasVollbrecht, JoachimKleinke, HolgerVollmer, MalteKleiziene, RitaVolochaev, V. A.Klemczak, BarbaraVolodin, Alexander M.Klemenc, JernejVoloshin, ArkadyKlimek, KatarzynaVolpe, MaurizioKlimkowicz-Pawlas, AgnieszkaVolpp, JoergKlimm, DetlefVon Gratowski, SvetlanaKliros, George S.Vona, MarcoKlishin, SergeyVondřejc, JaroslavKlobcar, DamjanVora, HiteshKlöcker, HelmutVora, AnkitKlontzas, MichaelVoronenkov, VladislavKlopf, John MichaelVorontsov, AlexanderKlose, ChristianVougioukas, EmmanouilKloukos, DimitriosVouros, IoannisKlusak, JanVoutetaki, Maria–StylianiKlyui, Nickolai I.Voznyak, YuriKmita, AngelikaVrabič Brodnjak, UrškaKnápek, AlexandrVrána, RadekKnop, YanivVranceanu, Diana M.Knyazeva, MarinaVrcek, Ivana VinkovicKnyziak, PiotrVrcelj, RankoKo, Yong-HoVrsaljko, DomagojKo, ChanghyunVryonides, PhotosKo, SeunghwanVukoje, MarinaKo, Dae-CheolVulin, DomagojKobata, JunpeiVuong, Thanh HuyenKobayashi, MakikoVuorimaa-Laukkanen, ElinaKobayashi, KayaVuye, CedricKoblischka, Michael R.Vyazovkin, SergeyKobtsev, SergeyVyskočil, VlastimilKocáb, DaliborVyšvařil, MartinKocharian, ArmenWachowski, MarcinKochetova, Nadezhda AlexandrovnaWadge, MatthewKochmańska, AgnieszkaWagh, PriyeshKochmański, PawełWagner, StefanKocich, RadimWainstein, DmitryKocjan, AndražWaite, Matthew M.Kockmann, NorbertWajda, AleksandraKoduru, JanardhanWalach, WojciechKoel, MihkelWalczak, RafałKoenders, E.A.B.Waldmann, DanièleKoenig, AndreasWałęsa-Chorab, MonikaKogia, MariaWallace, Kenneth N.Koh, Young-HagWalock, MichaelKoh, Yee RuiWalsh, Laurence J.Kohl, ThierryWalters, David J.Köhl, GudrunWalther, FrankKohlmann, HolgerWalvekar, Aditya A.Koike, MariWan, ShanhongKoike, RyoWanat, LeszekKoike, KenzoWanda, Wilczyńska-MichalikKoji, IchiiWang, YongxiangKojima, ToshikatsuWang, DanlingKojima, YoshiyukiWang, TingyunKokado, KentaWang, JunjieKokanyan, NinelWang, Shi-BinKokol, VanjaWang, HanlinKolasińska, EwaWang, ChuanlongKoletsi, DespinaWang, XuanKolev, SvetoslavWang, TianhaoKoleva, Iskra Z.Wang, ZhiguangKöller, ManfredWang, YuKolmas, JoannaWang, XiaoqingKolosnjaj-Tabi, JelenaWang, JianKoltunowicz, TomaszWang, YongKolubaev, Evgeny A.Wang, ZhejunKomarov, VictorWang, Xiao YongKomarov, PavelWang, ShifengKomarova, EkaterinaWang, ZhaoKomasa, SatoshiWang, JiaminKomissarov, AlexanderWang, XinQingKomolafe, AbiodunWang, XiaofengKonar, ArkaprabhaWang, PengtaoKondinski, AleksandarWang, ZhongchangKong, Xiang-TianWang, YuewuKong, Ling BingWang, TaoKong, FandeWang, HuKong, LingyunWang, Dung-AnKong, XiangxiongWang, LingKong, QingzhaoWang, LongKönigsberger, MarkusWang, LizhuKoniorczyk, MarcinWang, KunKonkol, JanuszWang, MingqingKonosu, ShinjiWang, ZhiKonovalov, SergeyWang, QinyiKonsulova-Bakalova, MariyaWang, YanshuaiKontonasaki, EleanaWang, FengwenKontoni, Denise-Penelope N.Wang, ZhenfengKontou, EviWang, ZhihanKontziampasis, DimitriosWang, KaiKoo, Bon HeunWang, XiaoenKopal, IvanWang, HaoyuKopeček, JaromírWang, ChengxinKopel, PavelWang, QunKopitar, DraganaWang, HuiKoplík, JanWang, FeiKoptyug, AndreyWang, Yun-CheKopyściański, MateuszWang, ChunjinKoral, CanWang, A-ChengKordas, KrisztianWang, ZhuqingKordesch, MartinWang, DaweiKorkmaz, KasimWang, GuiKorlos, ApostolosWang, BaoyuKorlyukov, Alexander A.Wang, YingKorneva, AnnaWang, YongqiangKorniejenko, KingaWang, HaopengKorosteleva, Elena N.Wang, XiaodongKoroteev, Pavel S.Wang, XiaoguangKorshunov, Maxim M.Wang, DuanyiKorsunsky, Alexander M.Wangler, Timothy P.Korzeniewska, EwaWan-Wendner, RomanKorzhikova-Vlakh, EvgeniaWarcholiński, BogdanKosec, TadejaWardeh, GeorgeKosec, BorutWarmuzek, MałgorzataKoshimizu, MasanoriWaryoba, Daudi R.Kosova, Nina V.Waseem, Owais AhmedKostecki, MarekWashio, AyakoKostiainen, MauriWasilewski, TomaszKostoglou, NikolaosWatanabe, MasahitoKostolný, IgorWatanabe, HiromichiKostryzhev, AndriiWatras, AdamKosturek, RobertWatzky, MurielleKosuke, NozakiWaugh, DavidKosyanov, DenisWawrzeńczyk, JerzyKoteš, PeterWeaver, JordanKotov, AntonWeber, FranzKotsiantis, SotirisWeber, IrisKotsiomiti, EleniWeems, Andrew C.Kotsuchibashi, YoheiWęglarski, MariuszKoubiti, MohammedWęglowski, MarekKoumoulis, DimitriosWęgrzyn, TomaszKoumoulos, Elias P.Wei, XialuKourousis, KyriakosWei, GangKousaka, HiroyukiWei, JianqiangKoushik, DibyashreeWei, DongbinKoutas, LamprosWei, KongchangKoutavarapu, RavindranadhWeis, MartinKoutiri, ImadeWeisheit, AndreasKoutny, DanielWeißensteiner, IrmgardKoutselas, IoannisWejrzanowski, TomaszKoutsovitis, PetrosWeller, Michael G.Koutzarova, TatyanaWellmann, PeterKouzu, MasatoWeltsch, ZoltánKováč, JaroslavWen, Amy M.Kovacevic, EvaWen, TongKovacik, JaroslavWen-Jeng, HoKovacs, GaborWertz, Philip W.Kovacs, LaszloWestermann, IdaKovács, TündeWetzel, BerndKovács, RóbertWexler, DavidKovács, ZsoltWheeler, RobertKoval, NikolayWhite, GregKovalchenko, Andrii M.Whitehead, Debra E.Kovaleva, MarinaWiącek, JoannaKovarik, OndrejWibowo, HartantoKovnir, KririlWick, Collin D.Kovvasu, Surya PrakasaraoWidbiller, MatthiasKowalak, StanisławWie, Jeong JaeKowalczuk, MarekWiecha, Peter R.Kowalczyk-Gajewska, KatarzynaWieckiewicz, MieszkoKowalska, EwaWieczorek, Władysław G.Kowalski, AleksanderWieczorowski, MichalKowalski, KamilWiegard, BjarneKowalski, KazimierzWieleba, WojciechKowalweska, AnnaWielgosz, MaciejKowerdziej, RafałWierzbicka-Miernik, AnnaKozak, YoramWierzbicki, MateuszKozdraś, AndrzejWieslawa, Nocun-WczelikKozhukhova, Natalia IvanovnaWilczyński, KrzysztofKoziol, MateuszWilding, MartinKoziol, KrzysztofWiles, AlanKoziorowska, AnnaWilk, JoannaKozlov, GlebWilke, OlafKozlowska, JustynaWilkins, Richard T.Kraft, ThomasWilkinson, Richard D. A.Kragović, MilanWille, SebastianKrahl, ThoralfWillenberg, BradleyKrajewski, PiotrWilliams, René M.Krajewski, WitoldWilsens, KarelKraker, ElkeWilthan, BorisKrakowiak, StefanWinczek, JerzyKrammer, OliverWinfree, Christopher J.Kranz, StefanWiniarski, JuliuszKratzer, MarkusWiniczenko, RadoslawKraus, ArminWinkler, AnjaKrause, Hans-JoachimWinkler, David A.Krause, Amanda R.Winlove, PeterKrawczyk, JacekWińska, KatarzynaKrawczyk, ŁukaszWinter, Benjamin D.Krawczyk, PiotrWinter, IanKreislova, KaterinaWinter, AlexanderKrejcar, OndrejWiraja, ChristianKrejsa, MartinWisner, BrianKrella, AlicjaWisniewski, AdamKrenner, HubertWitkowski, ArturKrishnamurthy, AjayWitt, KatarzynaKrivetskiy, ValeriyWitte, GregorKřivý, VitWitte, HartmutKrizan, PeterWittemann, FlorianKröger, RolandWittmann, Folker H.Krohns, StephanWlodarczyk, RenataKrol, JanWłodarczyk, PatrykKról, MariuszWohlert, JakobKról, MagdalenaWohlrab, SebastianKrólczyk, GrzegorzWojciechowski, ŁukaszKrólicka, AgnieszkaWojciechowski, SzymonKról-Kilińska, ŻanetaWójcik, GrzegorzKroll, PeterWojnarowicz, JacekKroner, Anna B.Wojtaszek, MarekKrsjak, VladimirWolanśki, WojciechKrstulović, NikšaWolarz, ErykKrstulović-Opara, LovreWołczyński, WaldemarKrueger, Joanna K.Wolf, ChristophKruk, AdamWolfe, Tonya B.Krumeich, FrankWöll, DominikKruppke, BenjaminWollweber, JuergenKrushynska, AnastasiiaWolny, StefanKruszewska, NataliaWolny, JuliuszKruszynski, RafalWolski, LukaszKrylov, Alexander S.Won, SoonhoKrymsky, Valeriy V.Wong, Roong JienKrystofiak, TomaszWong, BryanKrztoń-Maziopa, AnnaWong, Man-kinKrzywon, RafalWong, EugeneKrzyzanek, VladislavWoo, Yun SungKsiazek, MarzannaWood, DavidKtena, AphroditeWoszuk, AgnieszkaKu, ZahyunWoyciechowski, PiotrKu, Bon-CheolWozniak, JarosławKubasiewicz-Ross, PawelWrobel, RafałKubisztal, JulianWróbel, TomaszKubit, AndrzejWróblewski, RafałKubo, MasatakaWu, XijiaKučerová, LudmilaWu, Chia-WenKucharski, StanisławWu, XiangfaKuchler-Bopp, SabineWu, XuejianKucinska-Lipka, JustynaWu, Ming-ChungKucinski, KrzysztofWu, Shih-ChingKucuk, IsrafilWu, WeiKuczmaszewski, JozefWu, YaqiaoKúdela, JozefWu, HongjingKudelski, AndrzejWu, Tzi YiKudiiarov, ViktorWu, Zhong-ShuaiKudryashov, Sergey I.Wu, ChenshengKudryashova, OlgaWu, YunlongKugler, SzymonWu, GuoZhongKuhn, ErikWu, ShenghuaKujawa, JoannaWu, Hung-ChinKujawski, WojciechWu, HaoKukla, ChristianWu, Lyndia C.Kukle, SilvijaWu, Jing-YunKukli, KaupoWu, JiaminKukushkin, Sergey А.Wu, Jiunn-JongKula, KrzysztofWunderlich, WilfriedKulagin, RomanWuttke, StefanKulatilaka, WarunaWuu, Dong-SingKulichikhin, ValeryWychowaniec, JacekKulinich, SergeiWydrzyński, DawidKulka, MichalWyman, IanKulkarni, RomitWysokowski, MarcinKulkov, Sergei N.Wyszyński, DominikKulshreshtha, PrashantXavier Gil, JavierKumanek, BogumiłaXi, KaiKumar, HarishXia, GuanglinKumar, DushyantXia, ChangleiKumar, AvneeshXiang, PingKumar, PawanXianyu, YunleiKumar, VivekXiao, YueKumar, AnujXiao, GuijianKumar, VineetXiao, LiKumar, NarendraXiao, ZhigangKumar, ShailabhXie, YunchuanKumar, Kuppusamy SenthilXie, HaiboKumar, SamirXie, JingxiuKumar, PankajXie, ShangranKumar, SanjayXie, KunpengKumar, BrajeshXiong, RengenKumar, AlokXu, YitingKumar, NavinXu, TaoKumar Thakur, VijayXu, ZhiwuKumar Yadav, SusheelXu, Yi-JunKumeria, TusharXu, ChaoKundin, JuliaXu, ZheKundu, ArpanXu, YiKunetsov, VladimirXu, JiagangKunimine, TakahiroXu, LichongKuntz, MatthiasXu, JianKuo, Wen-TenXu, LongjunKuo, Jui-ChaoXu, WeiKuo, Che NanXu, JunhuaKuo, Pei-YuXu, GuKuok, Sin-ChiXu, ChunfuKuorwel, KuorwelXu, SongKuo-Yung, HungXuan, ShouhuKupai, JozsefXue, ChuangKurahashi, TakuyaYadav, HemrajKurczewska, JoannaYadav, SushmaKurda, RawazYadav, MithileshKureha, TakumaYadav, MarshleenKurek, AndrzejYadav, Hemraj MKuřitka, IvoYadavalli, Nataraja SekharKurlyandskaya, Galina V.Yagami, KimitoshiKuroki, HiroshiYaghoobi, MohammadrezaKurpinska, MarzenaYaghoubi, EhsanKurumisawa, KiyofumiYagi, MamikoKuryntsev, Sergey VyacheslavovichYagihara, ShinKurzina, Irina A.Yahiaoui, RiadKurzydłowski, Krzysztof J.Yakimov, E.B.Kushiro, TetsuoYakimova, LuidmilaKusiak-Nejman, EwelinaYakout, MostafaKusior, AnnaYakovenko, RomanKusiorowski, RobertYakushenko, AlexeyKustrowski, PiotrYamabe, JunichiroKut, StanisławYamabe-Mitarai, YokoKutikhin, Anton G.Yamada, ShuheiKuzhir, Polina P.Yamagishi, KentoKuzmicheva, AnastasiyaYamaguchi, SatoshiKuznetsov, SergeyYamamoto, GoKuznetsov, VyacheslavYamato, TakehikoKuznetsova, E. IrenYamato, MasafumiKuznetsova, LyubaYamauchi, YusukeKuźnicka, BogumiłaYan, XiangKuzuya, AkinoriYan, KaiKvande, ToreYan, BingxiKvapilova, MarieYan, WenyiKvasha, AndriyYan, YuKvashnin, Dmitry G.Yan, XueliangKwaśniak, PiotrYan, DingKwiatkowski, KonradYanase, KeijiKwiecień, IwonaYanaseko, TetsuroKwon, Jae SungYandouzi, MohammedKwon, Tae-YubYáñez-Vilar, SusanaKwon, Soon-HongYang, Ta-I.Kwon, Sung-NamYang, KejiaKwon, Ki-YoungYang, GuangweiKwon, IlKeunYang, Chung-WeiKwon, GibumYang, Yueh-Hsun KevinKyeongsik, WooYang, Jen-ChangKyratsis, PanagiotisYang, C.-F.Kyritsakis, AndreasYang, Chih-ChengKyzas, GeorgeYang, LeiKyziol, KarolYang, QuanlingLa, Duc DuongYang, JianxiaoLa Flor, Silvia DeYang, WeiLa Malfa Ribolla, EmmaYang, SungchulLa Mantia, Francesco PaoloYang, ZhiLa Rosa, GuidoYang, Tzu-SenLa Spada, LuigiYang, Hang-ShengLaakso, SampsaYang, JianminLaasri, SaidYang, ShoufengŁach, MichałYang, Su-ChulLachowicz, MarzenaYang, YingLachowicz, DorotaYang, LilyLacidogna, GiuseppeYang, Rong-SenLacki, PiotrYang, QuanlongLaefer, Debra F.Yang, GuangLago, Ana B.Yang, JingŁagoda, TadeuszYang, FanLaguna, Oscar H.Yang, Chih-YuLahouij, ImèneYang, RuihuaLai, Wen-FuYang, Seung-JaeLai, BinYang, XuLaity, Peter R.Yang, MinyangLaleh, MajidYangui, AymenLam, EdmondYao, TiankaiLamarre, Jean-MichelYao, HuiLambert, PaulYao, LanLambert, Charles-HenriYao, En-PingLamberti, LucianoYarema, KevinLambiase, FrancescoYasin, SohailLambin, PhilippeYavor, YinonLambrinou, KonstantinaYazdani Sarvestani, HamidrezaLamon, JacquesYazdi, ImanLampakis, DimitriosYe, AdamLampke, ThomasYe, MingxiaoLampropoulos, AndreasYe, YuLamy De La Chapelle, MarcYeager, JohnLan, Ting-HsunYeh, Chun-LiangLanba, AsheeshYehezkeli, OmerLancaster, RobertYeih, Wei-ChungLanceros-mendez, SenentxuYen, CheongLandi, MarcoYeo, In-SungLandowski, MichałYeo, JingjieLang, XiaolongYeo, HongLange, HeikoYeo, JunhoLanger, Jerzy JYeon, Kyu-SeokLanjewar, HarishchandraYeon, JaeheumLantsoght, EvaYeon, Jung HeumLanza, AntonioYeong, Wai YeeLanzerstorfer, ChristofYepes, VíctorLapčević, VeljkoYermán, LuisŁapińska, BarbaraYeung, Andy Wai KanŁapiński, MarcinYi, YougenLappa, IliadaYi, J.B.Laptev, AlexanderYifei, SunLaptev, RomanYifeng, LingLarciprete, RosannaYilmaz, MehmetLari, LeonardoYilmaz, ErolLarosa, ClaudioYio, MarcusLarsson, KarinYliniemi, JuhoLasheras, AndoniYokose, SatoshiLashgari, Hamid R.Yokota, ShingoLasik-Kurdyś, MałgorzataYoo, Dong JinLaskowska, MagdalenaYoo, JeeyoungLaskowski, ŁukaszYoo, ByungseokLasn, KasparYoon, Jin YoungLastovina, TatianaYoon, HyeonseokLaszcz, AmadeuszYoshida, SanichiroLates, Mihai-TiberiuYoshida, EriŁatka, LeszekYoshida, JunLatorre, MarcosYoshihara, HiroshiLauenstein, Jean MarieYou, LingyunLaurenti, MarcoYou, IlhwanLautner, GergelyYoung, DavidLaux, StevenYourey, WilliamLaux, Christoph J.Yousaf, JawadLavagna, LucaYousef, SamyLaviano, FrancescoYousefi, BardiaLaycock, BronwynYoussef, GeorgeLazar, IwonaYoussf, OsamaLázár, IstvánYu, XinjunLazarescu, LucianYu, ShengfuLazău, RaduYu, JiayiŁaźniewska-Piekarczyk, BeataYu, YunLazorenko, GeorgyYu, HaiLazpita, PatriciaYu, Tzyy LeonLazurenko, Daria V.Yu, JianLazzara, GiuseppeYu, HakkiLazzi, CamillaYu, ZiniuLe, HuirongYu, HaiboLe Borgne, BriceYu, WeilingLe Dréau, JérômeYucesan, GundogLe Febvrier, ArnaudYue, YuanLebeau, BénédicteYue, YanfengLebedev, Alexander AlexandrovichYuichi, SatoLebedev, Oleg IgorevichYun, Hyun DoLebon, FrédéricYun, Young SooLebon, NicolasYun, Seok JoonLebon, BrunoYung, Tung-YuanLebyodkin, Mikhaïl A.Yurchenko, OlenaLechner, Bob-DanYurchenko, NikitaLeclerc, CoreyYurkov, AndreyLecomte, Jean-SebastienYurukcu, MesutLecomte, PhilippeYusenko, KirillLedesma, Enrique FernandezYuya, SakaiLee, Sang JinZabala, AlaitzLee, Czang-HoZabierowski, PiotrLee, JaejunZabulionis, DariusLee, ChoongdoZacharatos, FilimonLee, Jin-KyunZafar, MuhammadLee, Sang-KonZagórski, IreneuszLee, Jae-UngZagulyaev, Dmitriy ValerievichLee, GeuntakZäh, MichaelLee, WonhyeZahran Al-Kamyani, ZahranLee, DonghyunZaidi, SaheemLee, Chang-JuZaidi, AliLee, WonyoungZaitsev, Alexandr A.Lee, DongkyuŻak, KrzysztofLee, GyoZakowski, KrzysztofLee, Young-ChulZamani, MazdakLee, Chia-HungZamanzade, MohammadLee, Sang-yupZambon, DanielLee, IlkeunZambon, AndreaLee, JaehyunZampieri, NicolòLee, Jong-JaeZandrini, TommasoLee, Han EolZanella, MattiaLee, Min-HungZanetti, ElisabettaLee, ChangwonZang, FahengLee, Joon KyuZangari, GiovanniLee, Yong-HakZani, GiulioLee, Hyun HoZanjani, Mehdi B.Lee, WookjinZannis, TheodorosLee, Bang YeonZanotti Fragonara, LucaLee, Chul-HeeZanotto, FedericaLee, MarkZappia, StefaniaLee, Jin WooZaquen, NeomyLee, SeunghoonZaraska, LeszekLee, Bor-ShiunnZareba, Jan K.Lee, GyudoZareiyan, BabakLee, VivianZarejousheghani, MashaalahLee, Hae-SeokZarkadoulas, AthanasiosLee, JeonghoonZarkevich, Nikolai A.Lee, YanZarogoulidis, PaulLee, Jung-MooZarrabeitia, MaiderLee, HwasungZarrelli, MauroLee, JoonseokZaucke, FrankLee, WonohZavašnik, JanezLee, Wen-JenZawada, KatarzynaLee, JongbeomZazpe, RaulLee, ByungjoonZdanowicz, MagdalenaLee, Young-JooZdarta, JakubLee, Du-HyeongZdarta, AgataLee, Seong-CheolZdenek, SloukaLee, Sun-KyuZdravecká, EvaLee, SeungaeZębala, WojciechLee, Ming-GinZebrev, GennadyLee, Shuo-JenZeeshan Mughal, MuhammadLee, JunseongZeidi, MahdiLee, Choon-manZeleny, ReinhardLee, Ik JinZelikman, EvgeniLeger, LilianeZeller-Plumhoff, BeritLegrand, VincentZeman, SvatoplukLehocky, MarianZendri, ElisabettaLei, YuchengZeng, ShuwenLei, YanhuaZeng, HulieLei, QianZeng, XiangkangLei, ZhenglongZeng, Jun-JieLeite, Fábio R.M.Žerjav, GregorLeitgeb, VerenaZervaki, Anna DemetriosLeitner, MichaelZet, CristianLeitner, MartinZgodavová, KristínaLejcek, PavelZhai, YuweiLekatou, AngelikiZhan, XiaowenLemos, José VieiraZhang, HongboLemr, KarelZhang, ZhienLemu, Hirpa G.Zhang, Sung-UkLen, ChristopheZhang, MengLenart, StanislavZhang, LijunLenzi, PaolaZhang, ZeyuLeonardi, RosaliaZhang, XiangLeonidas, PalilisZhang, ZhengjunLeonowicz, MarcinZhang, Xueqiang AlexLeonzio, GraziaZhang, ShanglongLeopold, ChristianZhang, XinchangLepadatu, DanielZhang, WeiLepicka, MagdalenaZhang, LaichangLepoittevin, BénédicteZhang, YuLeporatti, StefanoZhang, YufengLeś, AndrzejZhang, DiLesiuk, GrzegorzZhang, XianghuaLesov, IvanZhang, JianyingLesz, SabinaZhang, HangfengLeszczyńska-Madej, BeataZhang, ShuaifangLeszczynski, JuliuszZhang, YunyanLétoublon, AntoineZhang, AnqiLettieri, MariateresaZhang, ZhidongLeu, BogdanZhang, LiyuanLeung, Christopher K. Y.Zhang, GenLevartovsky, ShifraZhang, WenxuLeveneur, JeromeZhang, WeicaiLevine, MindyZhang, RujingLewandowski, MikołajZhang, QianLewis, GladiusZhang, WenjunLewis, RandolphZhang, YanlinLeżańska, MariaZhang, ChaoLezzerini, MarcoZhang, YaohongLgaz, HassaneZhang, Lai-ChangLi, SiminZhang, YongLi, XiaojunZhang, GuannanLi, Lu HuaZhang, PanpanLi, ShanshanZhang, DongzhouLi, Yi-Chen EthanZhang, ShunpingLi, JichaoZhang, DouLi, YingZhang, RuizhiLi, MeichunZhang, QichunLi, TaoZhang, HaiLi, ShiqiZhang, XinLi, JiantongZhang, WeilanLi, XinZhang, QiLi, Kuo-TsengZhang, JulieLi, PengZhang, XiaoLi, JiehuaZhang, TongshengLi, LiqunZhang, XuyangLi, Kai YuanZhang, JunjieLi, LingweiZhang, LuLi, ZhiZhao, GejianLi, MingchaoZhao, YufanLi, TanZhao, Evan WenboLi, JianZhao, XinxinLi, WeijieZhao, ShanLi, HengZhao, LianfengLi, Jiao JiaoZhao, LinpingLi, WenyueZhao, XiLi, Yeou-FongZhao, BoxuanLi, SinanZhao, WeiLi, WenxianZhao, YangLi, ZhenmingZhao, ChumingLi, HeZhao, YongxinLi, ZhaohuiZhao, YiliangLi, Hai-WenZhao, HongLi, YuanyuanZhao, WeijieLi, YangZhao, XinLi, ZhengweiZhao, YanyuLi, YiyanZhao, MingruiLi, XueminZhao, ShouLi, AnlongZhao, ZengfengLi, WeihanZhao, ZhiyongLi, YiZhen, ZhangLi, JinghaoZheng, KaiLi, WenyiZheng, BocongLi, YukunZheng, FeiLiang, ZhimingZheng, YiqunLiang, XiaopengZheng, XintingLiang, TianZherebtsov, SergeyLiang, Yuan-ChangZhidkov, Ivan S.Liang, SongmaoZhilyaev, AlexanderLiang, AnZhitova, ElenaLianos, PanagiotisZhong, TuhuaLiao, Shu-ChuanZhong, XiangyuLiao, Chien-SenZhong, MinlinLiao, KinZhou, YiqunLiao, Ying-ChihZhou, HongLiao, Wei SsuZhou, YangLiaw, Chya-YanZhou, XiaoyanLiberato, FerraraZhou, YajunLiberatore, Hannah K.Zhou, CaizhiLibessart, LaurentZhou, WeijieLichołai, LechZhou, YubinLichtenegger, HelgaZhou, AoLi-Destri, GiovanniZhou, ZhifuLiebman, JoelZhou, Xiao DongLienkamp, KarenZhou, ShengtaiLignola, Gian PieroZhou, JinmingLigon, Samuel ClarkZhou, YanboLikodimos, VlassiosZhou, ZhiLillo-Ródenas, Maria ÁngelesZhou, RanLim, SeokbinZhu, Shun-PengLim, SoomanZhu, HongtaoLim, Shuang FangZhu, GuanghuiLim, Dae-KwangZhu, LingxiangLim, BogyuZhuang, XiaoyingLim, DaewoonZhuravlev, VictorLim, OcktaeckZhuravleva, Tatyana S.Lim, KhoonŽic, MarkLim, Jae-HanZidek, JanLim, Ka RamZielinska, BeataLima, Bruno P.Zielińska-Jurek, AnnaLima, CarmineZielinski, AdamLimiti, ErnestoZieliński, AndrzejLin, GrayZiembowicz, SabinaLin, Hsin-TienZieniuk, BartłomiejLin, MaohuaZigdon-Giladi, HadarLin, JiaZigler, RadekLin, JianguoZille, AndreaLin, Wei-TingZima, AnetaLin, YuankunZima, BeataLin, Tz-FengZimnyakov, DmitryLin, HungyenZimoch-Korzycka, AnnaLin, JiahongZinigrad, MichaelLin, YuyuanZinoviev, AleksandrLin, Chia-HerZinovieva, OlgaLin, ZhiweiZiolkowski, RobertLin, Dan JaeZiora, ZytaLin, Chun-RongZkria, AbdelrahmanLin, JunrenŻmudzki, JarosławLin, Der-YuhZobel, PatrickLin, XiaoshanZoidis, PanagiotisLin, Tzu-EnZolfagharian, AliLin, MingyueZöllner, DanaLin, Chien-hongZolotoukhina, TatianaLin, Wen-ChinZolper, ThomasLin, WenyeŻółtowski, KrzysztofLindberg, GabriellaZornoza, EmilioLindemann, IngeZou, HaiyangLindner, MartinaZreiqat, HalaLinek, MałgorzataZubarev, AndreyLinert, WolfgangZubiaur, Pablo PérezLing, BoZubrzycki, JarosławLing, MengZuk, MagdalenaLingamdinne, Lakshmi PrasannaZukowski, WitoldLink, GuidoŽunda, AudriusLinko, VeikkoZuo, WenqiangLinul, EmanoilZuo, FeiLionetto, FrancescaZuo, LijianLiotier, Pierre-JacquesZuo, WenjieLisenkov, IvanZupančič, MatevžLisiecki, AleksanderZupanič, FrancLiška, MarekZuppinger, ChristianLisnevskaya, I.V.Zur, LidiaLitzius, KaiŻur, Krzysztof KamilLiu, Xian HuZwergel, ClemensLiu, MaoZych, TeresaLiu, PengfeiZygmunt, BogdanLiu, HongyuŻyła, GawełLiu, XifengŻyłka, ŁukaszLiu, ShuanglongZyuzin, Mikhail V.

